# Geochronology and geochemistry of granitic dikes in the Xiaogushan gold deposit NE China and implications for gold mineralization

**DOI:** 10.1371/journal.pone.0345886

**Published:** 2026-10-14

**Authors:** Wenzhao Fu, Yu Yang, Lei Zhu, Zhigang Yin, Lianfeng Gao, Zhenguo Zhang, Chao Liu

**Affiliations:** 1 College of Mining, Liaoning Technical University, Fuxin, Liaoning, China; 2 Liaoning Key Laboratory of Green Development of Mineral Resources, LNTU, Fuxin Liaoning, China; 3 College of Geosciences, China University of Petroleum, Beijing, China; 4 Suizhong Xinyuan Mining Co., Ltd., Suizhong, Liaoning, China; 5 Huludao Lianshi Chemical Co., Ltd., Huludao, Liaoning, China; National Autonomous University of Mexico Institute of Geophysics: Universidad Nacional Autonoma de Mexico Instituto de Geofisica, MEXICO

## Abstract

The Xiaogushan cryptoexplosive breccia-type gold deposit is located within the eastern Hebei-western Liaoning gold belt along the northern margin of the North China Craton. The mining area contains multiple episodes of subvolcanic dikes, among which the granite porphyry and granitic aplite dikes show close temporal and spatial relationship with gold mineralization. To constrain their emplacement age, petrogenesis, tectonic setting, and metallogenic significance, integrated zircon U-Pb-Hf isotopic and whole‑rock geochemical analyses were carried out on these dikes. Zircon U-Pb dating yields emplacement ages of 163.9 ± 2.0 Ma for the granite porphyry and 162.2 ± 4.9 Ma for the granitic aplite, indicating Late Jurassic magmatism in the region. The rocks are enriched in SiO_2_, K_2_O, and LILEs, depleted in HFSEs, and classified as strongly peraluminous, high‑K calc‑alkaline S‑type granites. The primary magma, derived from Paleoproterozoic lower crust (εHf(t) = −17.7 to −13.5; T_DM2_ = 2.29 to 2.07 Ga), and the geochemical variations are consistent with fractional crystallization of K‑feldspar and plagioclase. Regional tectonic evidence indicates that these dikes were emplaced in a continental collisional setting related to the closure of the Mongol‑Okhotsk Ocean. This collision caused crustal thickening, deformation, and subsequent extension, which promoted mineralization. These dikes characterized by high oxygen fugacity, alkali enrichment, and moderate evolution, may facilitate metal transport and remobilize pre‑existing mineralization via associated hydrothermal systems. Their clustered occurrence thus serves as a potential indicator for deep‑seated hydrothermal activity and gold mineralization, reflecting the intense Mesozoic tectono-magmatic-metallogenic event in the northern North China Craton.

## Introduction

The North China Craton (NCC) is the largest and oldest craton in China, with a nearly four billion years geological history [1–4]. Its tectonic evolution is multi-stage [5]. During the Archean (>2.5 Ga), the craton experienced the formation of continental nuclei and microblocks, followed by crustal accretion, culminating in initial cratonization at ~2.5 Ga through intense magmatic-metamorphic processes [2,3,6]. In the Proterozoic, the craton underwent tectonic superimposition of the Paleoproterozoic (2.3 ~ 1.9 Ga) active orogenic belt; at ~1.8 Ga, basement uplift and rift-non-orogenic magmatic events occurred, marking the final completion of cratonization [2,3,7]. From the Neoproterozoic to the Paleozoic, the NCC maintained a stable cratonic sedimentary cover [3]. During the Mesozoic, the NCC was successively subjected to subduction and compression from the South China Block to the south, the Siberian Plate to the north, and the Paleo-Pacific Plate to the east, resulting in extensive and intense tectonic activities along its southern, northern, and eastern margins [8]. The effects of these activities were not limited to the craton margins but extended into its interior, triggering large-scale magmatism, and ultimately leading to a transformation of the Mesozoic tectonic framework of the NCC, as well as the destruction and reconstruction of the craton [8,9]. Since the Cenozoic, its eastern margin has been further involved in the circum-Pacific tectonic system, becoming an important part of the active continental margin of Eurasian continent [3,10]. The tectonic evolution of the NCC directly controls regional gold mineralization, establishing it as China’s premier gold province, which contributes roughly 67% of national gold production and hosts numerous world-class gold deposits [11–14].

The western Liaoning Province is located on the northeastern margin of the North China Craton (Fig 1a). Due to its special tectonic position, it has witnessed and recorded the magmatic, tectonic and metallogenic processes in the northeastern part of the North China Craton since the Precambrian period [10,15–17]. As an important metallogenic cluster in the region, the eastern Hebei-western Liaoning gold metallogenic belt has excellent metallogenic conditions and has always been a key region for gold mineralization research [18–20]. Previous studies have demonstrated that gold deposits within the eastern Hebei-western Liaoning gold metallogenic belt exhibit a pronounced spatial and temporal correlation with granitoid intrusions [5,18,21–23]. Moreover, numerous gold deposits in this region are genetically associated with Mesozoic intermediate to felsic magmatic rocks, including intrusive bodies and dikes, which play a critical role in gold mineralization processes [21,22,24–27]. For instance, the Baizhangzi gold deposit, as one of the larger gold deposits on the metallogenic belt from eastern Hebei-western Liaoning, has a close relationship between gold mineralization and the Mesozoic granitic rock mass of the Baizhangzi [18,25]. The Shaoguoyingzi gold deposit in Jianping, western Liaoning, located in the south of the Shaoguoyingzi Jurassic biotite granite body, derives its ore-forming materials, heat sources, and ore-holding space from this granite body [28,29]. The formation of the large Yuerya gold deposit in eastern Hebei Province is genetically and spatially linked to the Yuerya granite, with both Mesozoic magmatic activity and gold mineralization primarily constrained to the 200 ~ 150 Ma interval [10]. The gold-polymetallic mineralization in the Tangzhangzi area of eastern Hebei is closely associated with the granite porphyry dikes in terms of both temporal-spatial distribution and genetic relationship, which is a response to the Mesozoic large-scale granite mineralization event in North China Craton [23,30–34].

**Fig 1:**
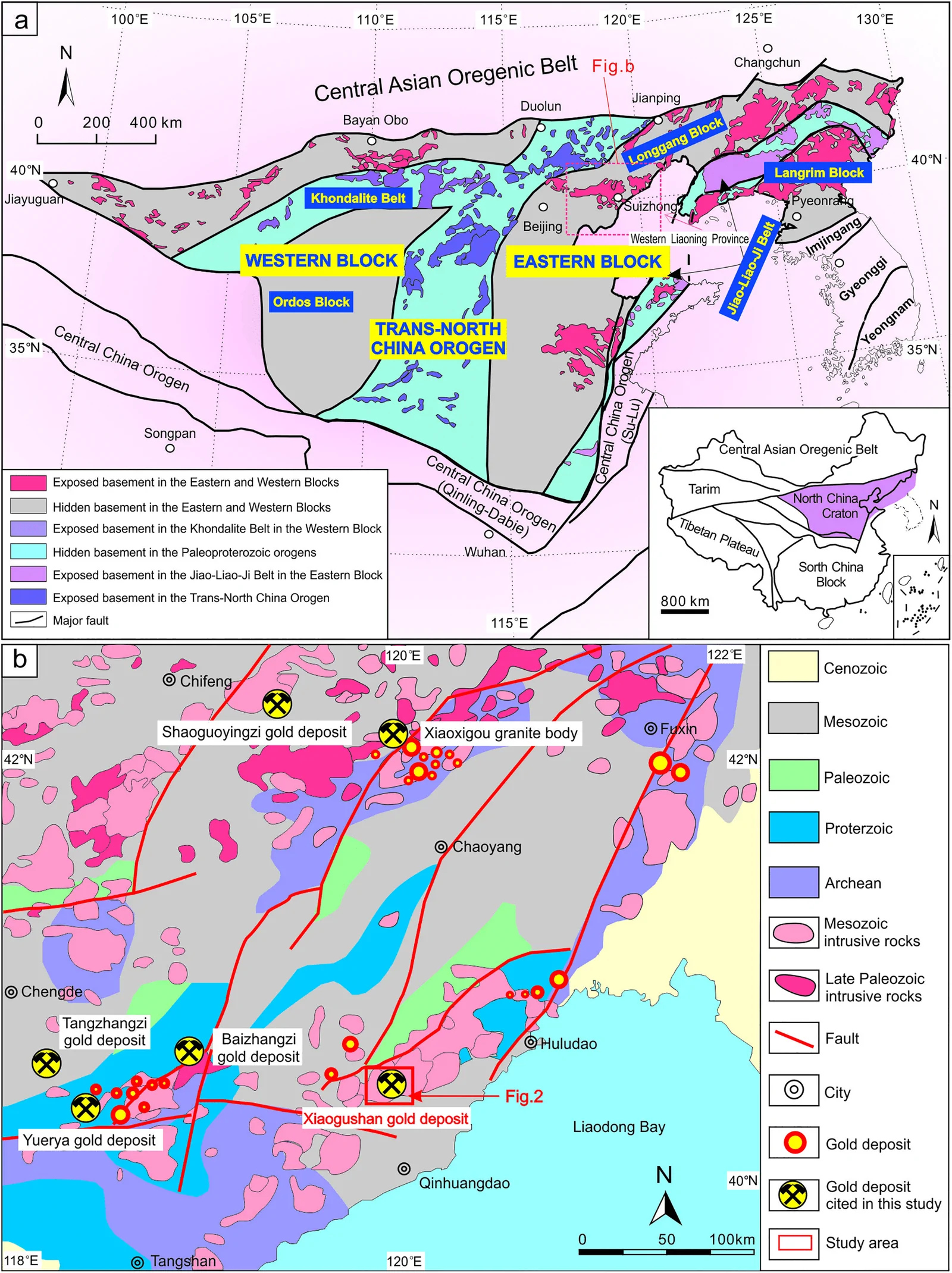
(a) Tectonic location of Xiaogushan gold deposit; (b) Geological map of eastern Hebei-western Liaoning, showing locations of Xiaogushan and several other gold deposits, including Baizhangzi, Tangzhangzi, Yuerya, Shaoguoyingzi and Xiaoxigou. Made with Natural Earth. Free vector and raster map data @ naturalearthdata.com.

The Xiaogushan gold deposit in Suizhong County, western Liaoning Province, NE China, located within the eastern Hebei-western Liaoning gold metallogenic belt, is classified as a small-scale gold deposit with proven gold reserves exceeding 3 tonnes (Fig 1b). The ore deposit is controlled by a cryptovolcanic breccia pipe hosted in Archean migmatitic granite, and is classified as a cryptoexplosive breccia-type gold deposit. The deposit area contains granite porphyry and granitic aplite dikes that strike consistently with the gold orebody. These dikes are products of regional tectono-magmatic activity, but their emplacement age, petrogenesis, tectonic setting, and relationship with gold mineralization remain unclear. Zircon U-Pb-Hf isotope and whole‑rock geochemical analyses were performed on these dikes to constrain their age, petrogenesis, tectonic setting, and links to gold mineralization. The results are of great scientific significance for constraining the mineralization age of the Xiaogushan gold deposit and for understanding its metallogenic evolution and genesis.

## Geological background

### Regional geology

Western Liaoning is located in the central segment of the northern margin of the North China Craton. It features well‑developed strata, complex structures, and intense Mesozoic magmatism, creating a favorable setting for gold mineralization and a key area for studying Mesozoic tectono‑magmatic processes [35–38]. The regional basement is composed of Archean to Paleoproterozoic metamorphic rock series, overlain by the Mesoproterozoic Changcheng and Jixian systems as sedimentary cover, with widespread Mesozoic volcano‑sedimentary strata [37,39]. This area has experienced multiple tectonic events, including Paleoproterozoic cratonization, Paleozoic-Mesozoic subduction-collision, and Yanshanian intracontinental orogeny, which together formed the NE‑ to NNE‑trending fault system, uplifts, and volcanic basins that control the distribution of gold belts [37,39,40]. Mesozoic granitic intrusions and volcanic rocks are widespread and closely associated with gold mineralization, supplying heat and ore-forming fluids [40–42].

### Deposit geology

The Xiaogushan gold deposit is located on the northern side of the Jiao‑Liao‑Ji orogenic belt, at the intersection of the Shanhaiguan paleo‑uplift and the western Liaoning depression (Fig 1). Its geographic coordinates are 120°00′58″E, 40°22′32″N (Fig 1b). The mining area exposes an Archean migmatitic granite basement and a volcanic apparatus filling sequence. The basement is dominated by Neoarchean monzogranite. The Xiaogushan cryptoexplosive breccia pipe intrudes into the basement, showing an intrusive‑cryptoexplosive contact. The pipe consists, from the outer to the inner part, of granitic agglomerate breccia, tuff breccia, and lithic‑crystal tuff. The lithic‑crystal tuff in the core is the main gold‑bearing ore, and its zircon U‑Pb age ranges from 2510.7 to 2508.9 Ma, indicating that the volcanic apparatus mainly formed in the Neoarchean [43] A total of seven major faults have been identified in the mining area: the southern F7 and the northern F1 faults show no significant correlation with gold mineralization. A relatively complete ring fault system is developed inside the volcanic apparatus, consisting of five major faults (F2-F6), among which F6 is clearly related to gold mineralization and is the main ore‑controlling fault in the area (Fig 2a).

**Fig 2:**
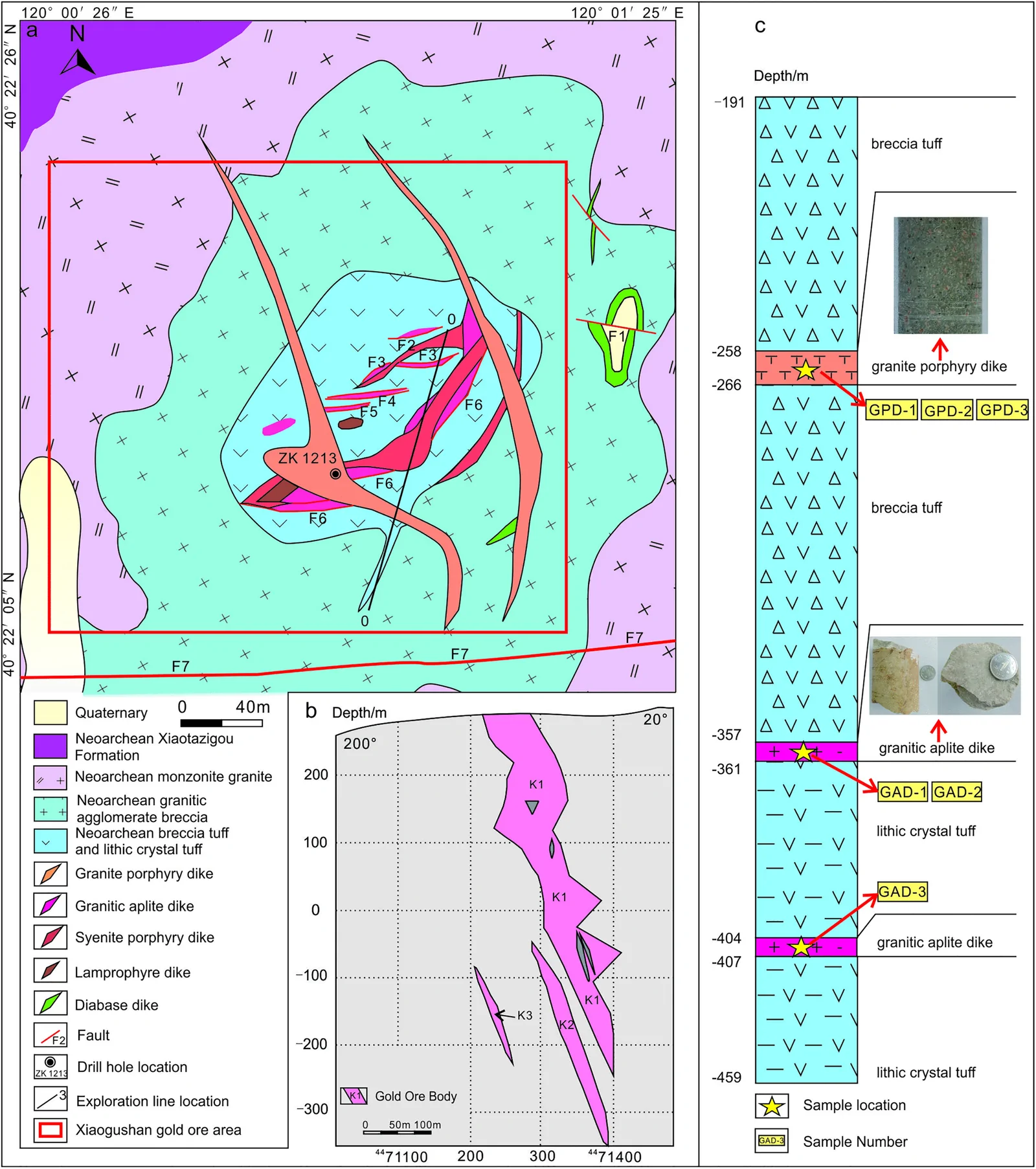
Geological maps and sections of the Xiaogushan gold deposit. (a) Regional geology; (b) Cross-section along Line 0; (c) Borehole ZK1213 profile.

Three gold ore bodies have been identified in the Xiaogushan gold deposit, and their spatial distribution is controlled by a cryptoexplosive breccia pipe located in the eastern part of the mining area (Fig 2b). The K1 gold ore body is an elliptical column, striking 290° and dipping 70° NE. K2, located in its footwall, occurs a thick vein striking 290° and dipping 68° NE. K3 is situated in the footwall of K2 as a vein, striking 55° and dipping 65° NW. The ore mineralization type is disseminated sulfide type, exhibiting hypidiomorphic-xenomorphic granular texture and disseminated structure. The metallic minerals mainly consist of pyrite, chalcopyrite, galena, sphalerite, specularite, magnetite, native gold, and native silver. The gangue minerals are predominantly quartz, K-feldspar, sericite, chlorite, epidote, and calcite. The main types of wall-rock alteration are silicification, potassic alteration, chloritization, and sericitization.

Sub-volcanic dikes occur in the area, comprising two NW-trending granite porphyry dikes (length >1,000 m, width 10 ~ 20 m) and a set of NE-trending granitic aplite dikes within and around the cryptoexplosive breccia pipe (discontinuously extending ~ 600 m, width 10 ~ 60 m) (Figs 2a and 3). Field observations show that the dikes exhibit an intrusive contact relationship with the strata in the mining area (Figs 3a and 3c). The dikes are consistent in trend with the orebody strike, and the orebodies are mostly concentrated near the dikes, with some gold orebodies crosscut by the dikes. The contact zones between the dikes and the wall rocks, as well as their surrounding areas, commonly exhibit alteration and mineralization assemblages, such as pyritization, silicification, and sericitization. These geological features suggest that the emplacement of the granitic dikes in the mining area likely postdates the main mineralization stage of the Xiaogushan gold deposit.

**Fig 3:**
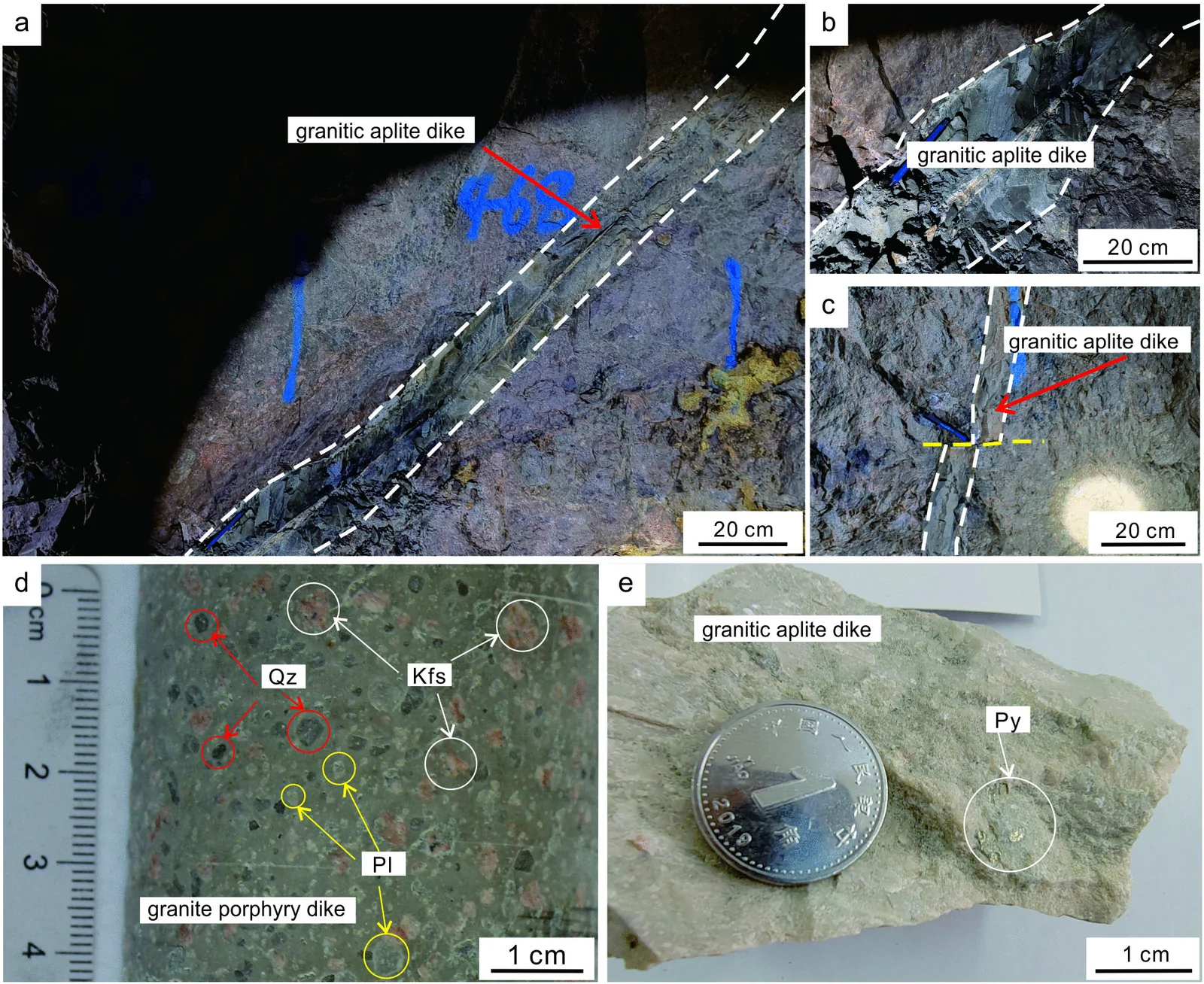
Field and collected specimen characteristic photos of granite porphyry dikes and granitic aplite dikes. (a-c) Field photograph of granitic aplite dikes cut through the orebody; (d) Hand specimen photograph of granitie porphyry dikes; (e) Hand specimen photograph of pyritized granitic aplite dikes. Py-pyrite; Qz-quartz; Kfs-feldspar; Pl-plagioclase.

## Samples and analytical methods

### Sample description

To obtain representative primary magmatic information, drill hole ZK1213 was selected for sampling because it intersects both major dike types and preserves the most complete geological sequence in the deposit (specific sampling locations are shown in Fig 2). Given the hydrothermal nature of the system, we selected the least altered samples and calculated the CIA to quantitatively evaluate alteration effects on mobile elements, thus enhancing the robustness of geochemical interpretations. Whole‑rock major and trace elements were analyzed for six dike samples (three granite porphyry: GPD 1–3; three granitic aplite: GAD 1–3). Zircon LA-ICP-MS U-Pb dating and in-situ Hf isotopes analysis were performed on GPD-2 and GAD-2 to constrain magmatic timing and source characteristics.

The granite porphyry samples are grayish‑green in colour and exhibit a porphyritic texture and massive structure (Fig 3d). The phenocryst content is approximately 50%, consisting of K‑feldspar (20%), plagioclase (15%), quartz (10%), and minor biotite (<5%) (Figs 4a and 4b). K‑feldspar occurs as subhedral to anhedral grains, 0.5 ~ 6 mm in size. Plagioclase occurs as subhedral to anhedral tabular crystals, 0.2 ~ 5 mm in size, with well‑developed polysynthetic twinning and local sericitization. Quartz occurs as subhedral to anhedral grains, 0.2 ~ 4 mm in size. Biotite is flaky, 0.1 ~ 4 mm in size. The matrix constitutes about 50% of the rock and exhibits a microcrystalline texture, composed mainly of microcrystalline quartz, K‑feldspar, plagioclase, and biotite. Accessory minerals include zircon, apatite, sphene and magnetite.

**Fig 4:**
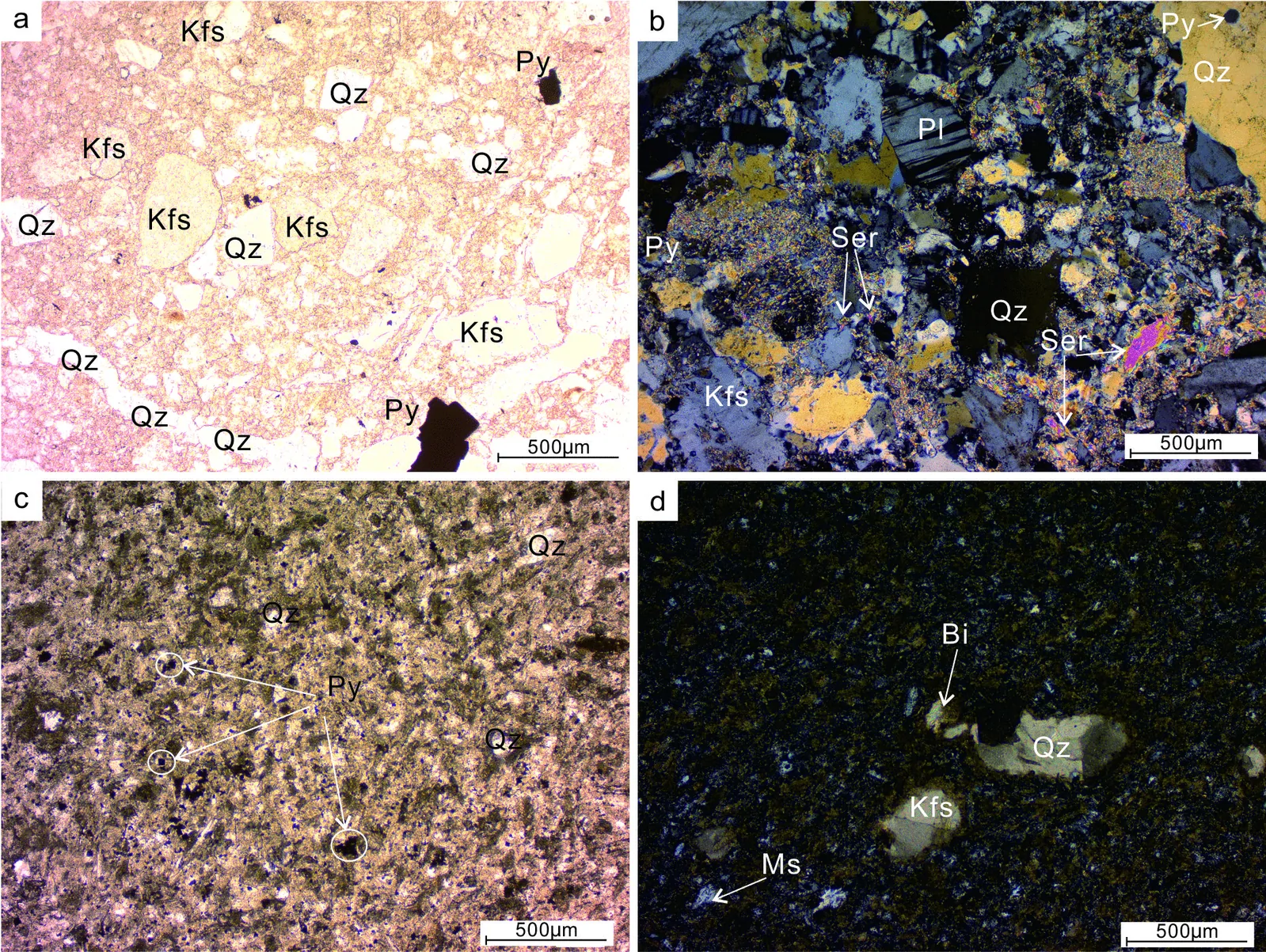
Photomicrographs of samples from the Xiaogushan gold deposit. (a) Granite porphyry dike sample containing a quartz vein; (b) Granitic aplite showing local sericitization of K-feldspar; (c-d) Granitic aplite dikes. Qz: quartz; Kfs: K-feldspar; Py: pyrite; Pl: plagioclase; Bi: Biotite; Ms: Muscovite.

The granitic aplite samples are grayish-white in colour and exhibit an aplitic texture and massive structure (Fig 3e). The main minerals are quartz (30 ~ 35%), plagioclase (25 ~ 30%), and K-feldspar (10 ~ 15%), with minor amounts of muscovite (<5%) and biotite (<5%) (Figs 4c and 4d). The mineral grains are fine, generally less than 100 μm in size. Quartz is mostly anhedral granular, filling the spaces between feldspar grains. Plagioclase occurs as subhedral tabular crystals with well-developed polysynthetic twinning. K-feldspar includes orthoclase and microcline, occurring as subhedral to anhedral grains; microcline commonly exhibits cross-hatched twinning. Biotite and muscovite are sparsely distributed as flakes. Accessory minerals include zircon, apatite, sphene and magnetite.

### LA-ICP-MS zircon U-Pb dating

Zircon sample preparation and isotopic analyses were performed at Beijing Zirconium Year Pilot Technology Co., Ltd. Rock samples were initially crushed, and zircon grains were separated using conventional heavy liquid and magnetic techniques. The selected zircon grains were mounted in epoxy resin and polished to expose internal structures. Cathodoluminescence (CL) imaging was then performed using IT-500 and DELMIC CL systems to reveal internal structures and select optimal analytical spots. U-Pb dating and trace element analyses were conducted using the NWR193UC 193 nm deep-UV laser ablation system coupled with the Agilent 7900 ICP-MS. Detailed instrumental parameters and tuning methodologies are provided in Reference [44]. Prior to analysis, the instrument was optimized using NIST SRM 610 and 612 standard glasses. Zircon standard 91500 was used as the primary reference material for U-Pb isotopic fractionation correction, and Plesovice (PLE) zircon was employed as a secondary monitor (for every 10 sample spots, two analyses of 91500 and one analysis of PLE were measured). Raw data were processed using ICPMSDataCal 7.7 software for background subtraction, isotopic ratio calculation, and common Pb correction [45,46]. Final age calculations and Concordia diagrams were generated using Isoplot [47].

### Zircon Lu-Hf isotope analyses

Zircon grains for Lu-Hf isotope analysis were selected based on the same spots or domains that yielded concordant U-Pb ages. The analyses were performed at Beijing Zirconium Year Pilot Technology Co., Ltd., using a Neptune Plus MC-ICP-MS equipped with a RESOlution SE 193 nm laser ablation system. Instrumental conditions, data acquisition and analytical processes followed references [48,49]. The decay constant of ^176^Lu is 1.867 × 10^-11^, and chondritic values of ^176^Hf/^177^Hf = 0.282772 and ^176^Lu/^177^Hf = 0.0332 were adopted to calculate εHf values [50]. One-stage model ages (T_DM1_) were calculated using present depleted mantle values of ^176^Lu/^177^Hf = 0.0384 and ^176^Hf/^177^Hf = 0.28325. Two-stage model ages (T_DM2_) were calculated using f _Lu/Hf (DM)_ = 0.15688 and f _Lu/Hf(CC)_ = −0.54833 [51,52].

### Whole-rock major and trace element analyses

Whole‑rock major and trace element analyses were performed at Beijing Zirconium Year Pilot Technology Co., Ltd. Major elements were determined using an Axiosmax X‑ray fluorescence spectrometer (XRF), with analytical precision better than 5%. Rock samples were first crushed to <200 mesh, then mixed thoroughly with Li_2_B_4_O_7_ and LiBO_2_ as fluxes. The mixture was fused at 1050 °C to produce homogeneous glass beads, which were subsequently analyzed by the Axiosmax XRF. FeO was determined by titration, and the loss on ignition (LOI), H_2_O^+^ and H_2_O^-^ contents were measured gravimetrically using a P124S balance. Trace elements were determined using an ICAPQ inductively coupled plasma mass spectrometer (ICP‑MS), with analytical precision better than 5%. Approximately 50 mg of sample powder was placed in a sealed digestion vessel, to which 1 mL HF and 0.5 mL HNO_3_ were added, and the mixture was heated for 24 h under sealed conditions. After cooling, another 0.5 mL of HNO_3_ was added and the solution was evaporated to near dryness; this step was repeated once. Then, 5 mL of HNO_3_ was added, and the vessel was resealed and heated for another 3 h. After cooling, the solution was diluted with water for ICP‑MS measurement. The detailed analytical procedures followed the Chinese National Standard GB/T14506‑2010 (Methods for chemical analysis of silicate rocks) [53].

## Results

### LA-ICP-MS zircon U-Pb dating

Zircon U-Pb dating and trace element results are presented in S1 and S2 Tables. Zircons from both dike types (GPD-2 and GAD-2) are 60 ~ 150 μm in size with aspect ratios of 1:1–3:1(Figs 5a and 5c). Cathodoluminescence images show euhedral crystals with oscillatory zoning, confirming a magmatic origin. Their chondrite-normalized REE patterns exhibit LREE depletion, HREE enrichment, positive Ce anomalies, and negative Eu anomalies (Figs 5b and 5d), consistent with typical magmatic zircon signatures [55,56].

**Fig 5:**
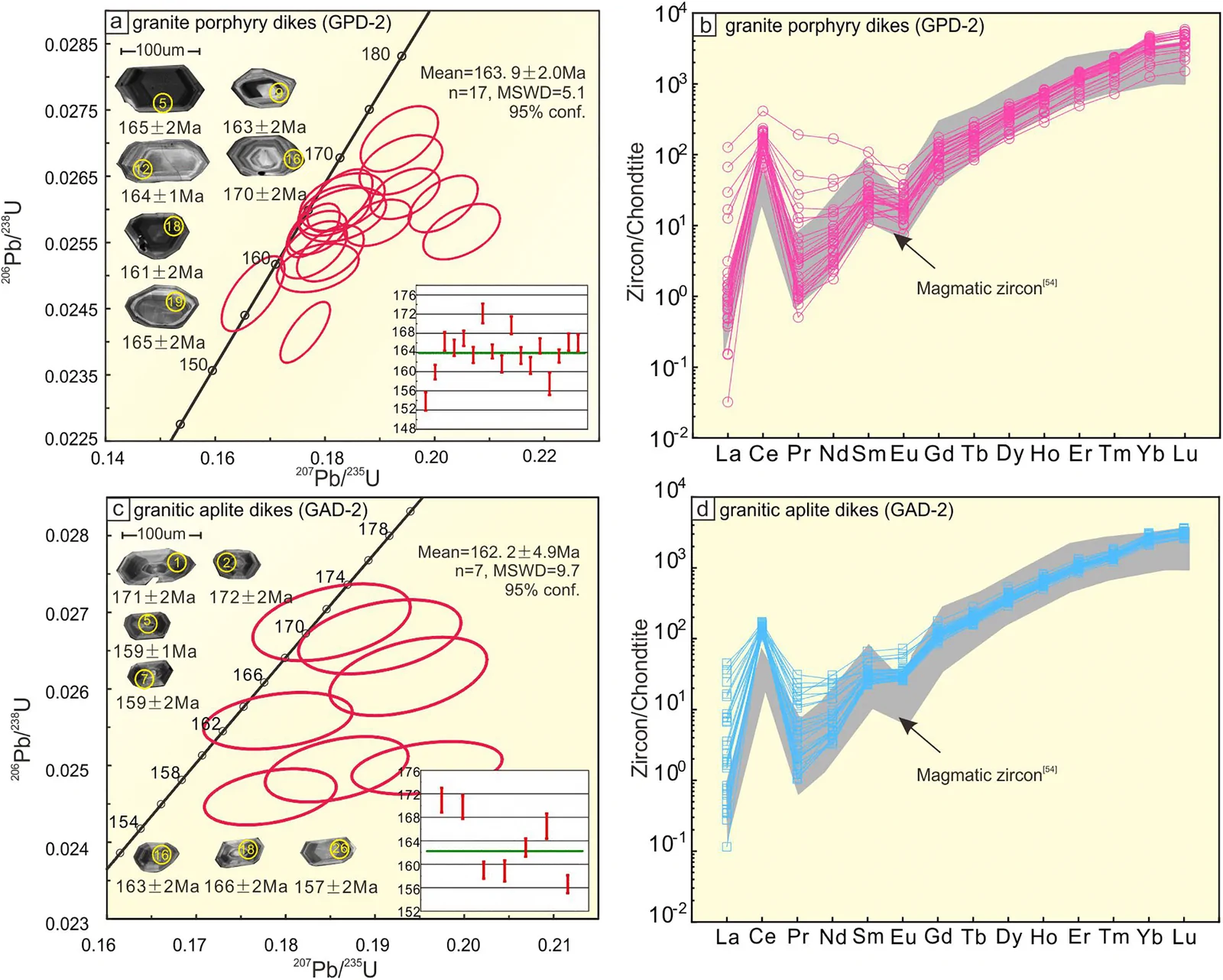
Zircon LA-ICP-MS U-Pb concordia diagrams, weighted average age diagrams, cathodoluminescence images of representative zircons and zircon chondrite-normalized REE patterns from the Xiaogushan gold deposit. (a-b) Granite porphyry dikes (sample No. GPD-2); (c-d) Granitic aplite dikes (sample No. GAD-2). The chondrite values are from Boynton (1984) [54].

Twenty-six zircon analyses on sample GPD-2 yielded Th (143 ~ 3529 ppm) and U (175 ~ 2028 ppm), with Th/U ratios of 0.76 ~ 1.74 (>0.1), consistent with a magmatic origin [57,58]. Nine zircon analyses yielded significantly older ^207^Pb/^206^Pb ages (2590 ± 90 Ma to 557 ± 59 Ma), indicating the presence of inherited zircons. The remaining seventeen analyses all fall on or below the concordia line, yielding a weighted mean ^206^Pb/^238^U age of 163.9 ± 2.0 Ma (MSWD = 5.1, Fig 5a).

Zircons from sample GAD-2 also exhibit magmatic signatures, with Th/U ratios of 0.89 ~ 1.57 (Th: 291 ~ 886 ppm; U: 311 ~ 616 ppm). Twenty-three of the thirty analyses yielded significantly older ^207^Pb/^206^Pb ages (ranging from3724 ± 210 Ma to 656 ± 70 Ma), reflecting the crystallization of inherited zircons. The remaining seven analyses all fall on or below the concordia line, yielding a weighted mean ^206^Pb/^238^U age of 162.2 ± 4.9 Ma (MSWD = 9.7, Fig 5c).

### Zircon Hf isotopes

Based on the zircon U-Pb results, 17 zircon spots were selected for Hf isotopic composition analysis, of which 10 were from the granite porphyry dike (GPD-2) and 7 from the granitic aplite dike (GAD-2). Zircon Hf isotope results are presented in Table 1. The ^176^Lu/^177^Hf ratios of the GPD-2 sample range from 0.001657 to 0.002543, with corresponding εHf(t) values of −16.36 to −13.47 (mean: −15.05) and T_DM2_ model ages of 2245 ~ 2066 Ma. Zircons from sample GAD-2 yield ^176^Lu/^177^Hf ratios between 0.001147 and 0.002185, εHf(t) values of −17.66 to −15.26 (mean: −16.44), and T_DM2_ ages of 2293 ~ 2180 Ma. The T_DM2_ ages derived from zircon Lu-Hf isotopes for both samples are significantly older than their respective crystallization ages.

**Table 1:** In situ zircon Hf isotopic data for granite porphyry dikes and granitic aplite dikes in Xiaogushan gold deposit.

Spots	Age (Ma)	^176^Yb/^177^Hf	2σ	^176^Lu/^177^Hf	2σ	^176^Hf/^177^Hf	2σ	ε_Hf_(0)	ε_Hf_(t)	T_DM1_ (Ma)	T_DM2_ (Ma)
GPD-2 (granite porphyry dikes)											
2	160	0.050639	0.000876	0.001952	0.000008	0.282252	0.000018	−18.39	−15.09	1447	2164
5	165	0.055155	0.000500	0.002145	0.000019	0.282296	0.000019	−16.85	−13.47	1392	2066
7	167	0.045859	0.001391	0.001710	0.000015	0.282258	0.000016	−18.16	−14.69	1428	2145
9	163	0.040890	0.001318	0.001628	0.000027	0.282262	0.000015	−18.04	−14.63	1420	2139
12	164	0.053675	0.001755	0.001998	0.000031	0.282237	0.000016	−18.94	−15.55	1471	2197
15	162	0.069048	0.000753	0.002543	0.000027	0.282217	0.000016	−19.63	−16.36	1521	2245
17	163	0.042192	0.001258	0.001547	0.000024	0.282246	0.000019	−18.61	−15.19	1440	2174
18	161	0.045961	0.000458	0.001935	0.000017	0.282259	0.000017	−18.13	−14.80	1436	2147
19	165	0.047772	0.000530	0.001866	0.000012	0.282254	0.000017	−18.30	−14.88	1440	2155
22	163	0.045298	0.001242	0.001657	0.000032	0.282229	0.000019	−19.19	−15.79	1467	2211
GAD-2 (granitic aplite dikes)											
1	171	0.042046	0.002331	0.001447	0.000049	0.282190	0.000032	−20.58	−16.99	1515	2293
2	170	0.058842	0.001132	0.002185	0.000028	0.282174	0.000025	−21.13	−17.66	1568	2333
5	159	0.037611	0.000742	0.001453	0.000022	0.282223	0.000020	−19.41	−16.07	1468	2226
7	159	0.042891	0.000583	0.001656	0.000031	0.282221	0.000020	−19.48	−16.17	1479	2232
16	163	0.043417	0.000292	0.001695	0.000030	0.282213	0.000027	−19.78	−16.39	1493	2249
18	166	0.043590	0.000692	0.001595	0.000022	0.282242	0.000022	−18.73	−15.26	1447	2180
26	157	0.043336	0.001127	0.001687	0.000024	0.282211	0.000023	−19.82	−16.57	1494	2255

### Whole-rock geochemistry

The major and trace element compositions are presented in Table 2. The granite porphyry dike samples exhibit high SiO_2_ (68.18 ~ 73.46 wt%), K_2_O (8.14 ~ 8.73 wt%), Al_2_O_3_ (13.05 ~ 15.54 wt%), and low Na_2_O (0.21 ~ 0.44 wt%), MgO (0.28 ~ 0.74wt%), CaO (0.7 ~ 2.14 wt%) and TFe_2_O_3_ (0.96 ~ 2.50 wt%). In the TAS (Total Alkali-Silica) diagram (Fig 6a), all three samples plot in the granite field. In the K_2_O vs. SiO_2_ diagram (Fig 6b), samples plot in the shoshonitic field. The samples are peraluminous (A/CNK = 1.15 ~ 1.17) and plot in the peraluminous field on the A/NK vs. A/CNK diagram (Fig 6c). In the Na_2_O+K_2_O-CaO vs. SiO_2_ diagram (Fig 6d), all samples plot in the alkali-calcium field. The samples contain 74.52 ~ 130.07 ppm ΣREE (Total Rare Earth Elements), with chondrite-normalized (La/Yb)_N_ ratios of 24.09 ~ 32.79 indicating strongly fractionated, LREE (Light Rare Earth Elements) enriched patterns (Fig 7a). The δEu values range from 0.79 to 1.10, exhibiting both weakly negative and weakly positive Eu anomalies. All samples show enrichment in large ion lithophile elements (LILEs; e.g., Rb, Ba, K) but depletion in high field strength elements (HFSEs; e.g., Nb, Ta, Ti, P) on primitive-mantle-normalized trace element diagram (Fig 7b).

**Table 2:** Major (wt%) and trace elements (ppm) data for granite porphyry dikes and granitic aplite dikes in Xiaogushan gold deposit.

Lithology	granite porphyry dikes			granitic aplite dikes		
Sample	GPD-1	GPD-2	GPD-3	GAD-1	GAD −2	GAD −3
SiO_2_	68.18	73.46	73.14	78.13	76.38	76.11
TiO_2_	0.26	0.11	0.12	0.08	0.09	0.10
Al_2_O_3_	15.54	13.05	13.44	11.61	11.52	12.49
Fe_2_O_3_	1.38	0.59	0.55	0.42	0.48	0.18
FeO	1.01	0.46	0.37	0.22	0.30	0.14
MnO	0.05	0.10	0.07	0.01	0.09	0.02
MgO	0.74	0.44	0.28	0.27	0.21	0.14
CaO	2.14	0.76	1.23	0.12	0.24	0.07
Na_2_O	0.44	0.21	0.29	0.13	0.24	0.26
K_2_O	8.15	8.73	8.14	7.70	9.12	9.55
P_2_O_5_	0.14	0.03	0.03	0.01	0.01	0.01
H_2_O^+^	1.00	1.02	1.35	0.94	0.74	0.68
H2O-	0.22	0.15	0.31	0.25	0.28	0.27
LOI	1.58	1.93	2.21	1.13	1.10	0.73
Total	99.73	99.92	99.91	99.83	99.84	99.81
TFe_2_O_3_	2.50	1.11	0.96	0.66	0.82	0.33
K_2_O/Na_2_O	18.52	42.49	27.94	60.98	37.73	37.06
Na_2_O+K_2_O	8.59	8.94	8.43	7.82	9.37	9.81
A/NK	1.62	1.33	1.44	1.36	1.12	1.16
A/CNK	1.15	1.17	1.16	1.32	1.07	1.14
δ	2.93	2.62	2.36	1.74	2.63	2.91
Mg^#^	36.90	44.20	36.60	44.60	33.80	45.20
DI	92.80	93.40	93.70	94.40	96.30	96.60
AR	5.47	6.78	6.12	5.33	11.05	8.56
CIA	60.2	56.5	57.7	57.1	52.4	53.3
Rb	73.7	230.00	301.00	222.00	215.00	254.00
Ba	1269.00	474.00	454.00	1019.00	974.00	985.00
Th	3.35	8.14	10.4	5.07	6.09	6.98
U	0.75	2.41	3.04	1.39	3.07	3.71
Nb	8.02	9.16	11.7	6.92	8.44	8.21
Ta	0.55	0.97	0.76	0.61	0.64	0.57
Sr	591.00	142.00	91.2	131.00	146.00	132.00
Zr	118.00	79.4	95.6	79.4	87.6	102.00
Hf	2.93	2.85	3.53	2.61	2.90	3.28
Y	7.77	4.92	5.31	3.23	6.77	4.73
La	35.5	21.8	25.5	16.1	32.0	26.7
Ce	54.6	35.4	40.7	27.3	55.2	45.5
Pr	6.34	3.04	3.46	2.49	5.03	4.07
Nd	23.2	9.49	10.8	8.11	16.9	13.6
Sm	3.28	1.28	1.31	1.15	2.43	1.69
Eu	1.06	0.32	0.33	0.46	0.80	0.51
Gd	2.63	1.14	1.26	0.95	2.02	1.48
Tb	0.32	0.13	0.14	0.11	0.24	0.15
Dy	1.30	0.57	0.65	0.48	1.05	0.70
Ho	0.26	0.16	0.16	0.13	0.23	0.17
Er	0.64	0.40	0.44	0.31	0.61	0.48
Tm	0.12	0.094	0.10	0.074	0.13	0.10
Yb	0.73	0.61	0.71	0.47	0.79	0.68
Lu	0.091	0.086	0.11	0.054	0.11	0.090
∑REE	130.07	74.52	85.67	58.19	117.54	95.92
LREE	123.98	71.33	82.10	55.61	112.36	92.07
HREE	6.09	3.19	3.57	2.58	5.18	3.85
LREE/HREE	20.35	22.36	23.00	21.57	21.69	23.91
(La/Yb)_N_	32.79	24.09	24.21	23.09	27.31	26.47
(La/Sm)_N_	6.81	10.71	12.24	8.81	8.28	9.94
(Gd/Lu)_N_	3.59	1.65	1.42	2.19	2.28	2.04
δEu	1.10	0.81	0.79	1.35	1.10	0.99
δCe	0.88	1.05	1.04	1.04	1.05	1.05

Note: CIA = [Al_2_O_3_/ (Al_2_O_3_ + CaO* + Na_2_O + K_2_O)] × 100 (molar). CaO* = CaO if CaO ≤ Na_2_O; otherwise CaO* = Na_2_O. $\delta Eu=\frac{{Eu}_{N}}{\sqrt{{Sm}_{N}\times {Gd}_{N}}}$, $\delta Ce=\frac{{Ce}_{N}}{\sqrt{{La}_{N}\times {Pr}_{N}}}$.

**Fig 6:**
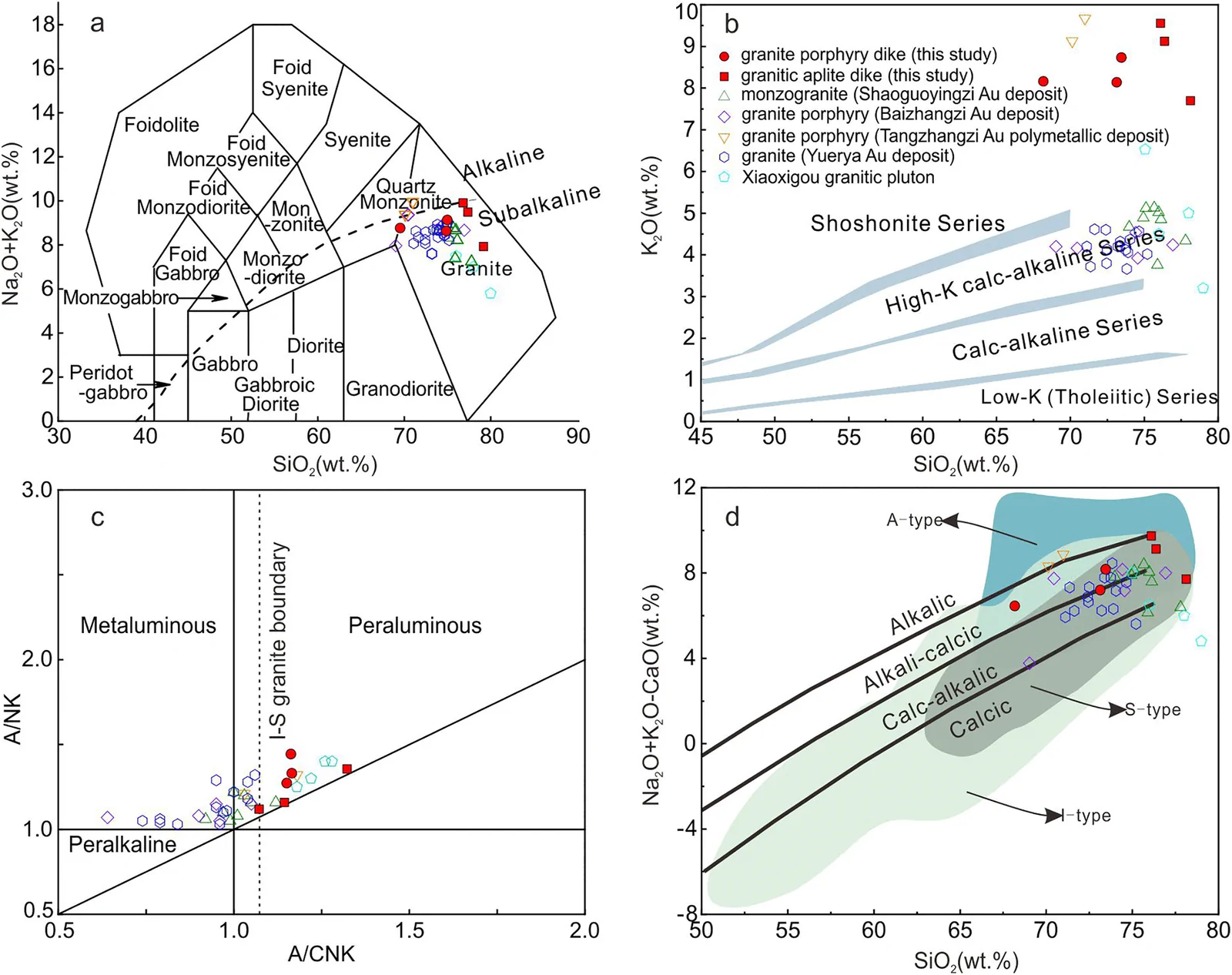
Diagrams of (a) Na_2_O+K_2_O vs. SiO_2_ (after Middlemost, 1994 [59]), (b) K_2_O vs. SiO_2_ (after Rickwood, 1989 [60]), (c) A/NK vs. A/CNK (after Maniar and Piccoli, 1989 [61]) and (d) Na_2_O+K_2_O-CaO vs. SiO_2_ (after Frost et al., 2001 [62]) for the granite porphyry dikes and granitic aplite dikes in the Xiaogushan gold deposit. The data for Shaoguoyingzi, Baizhangzi, Tangzhangzi, Yuerya and Xiaoxigou shown for comparison are from Chen et al. (2016) [29], Li et al. (2024) [18], He (2016) [30], Liu et al. (2015) [10] and Yang et al. (2019) [63], respectively.

**Fig 7:**
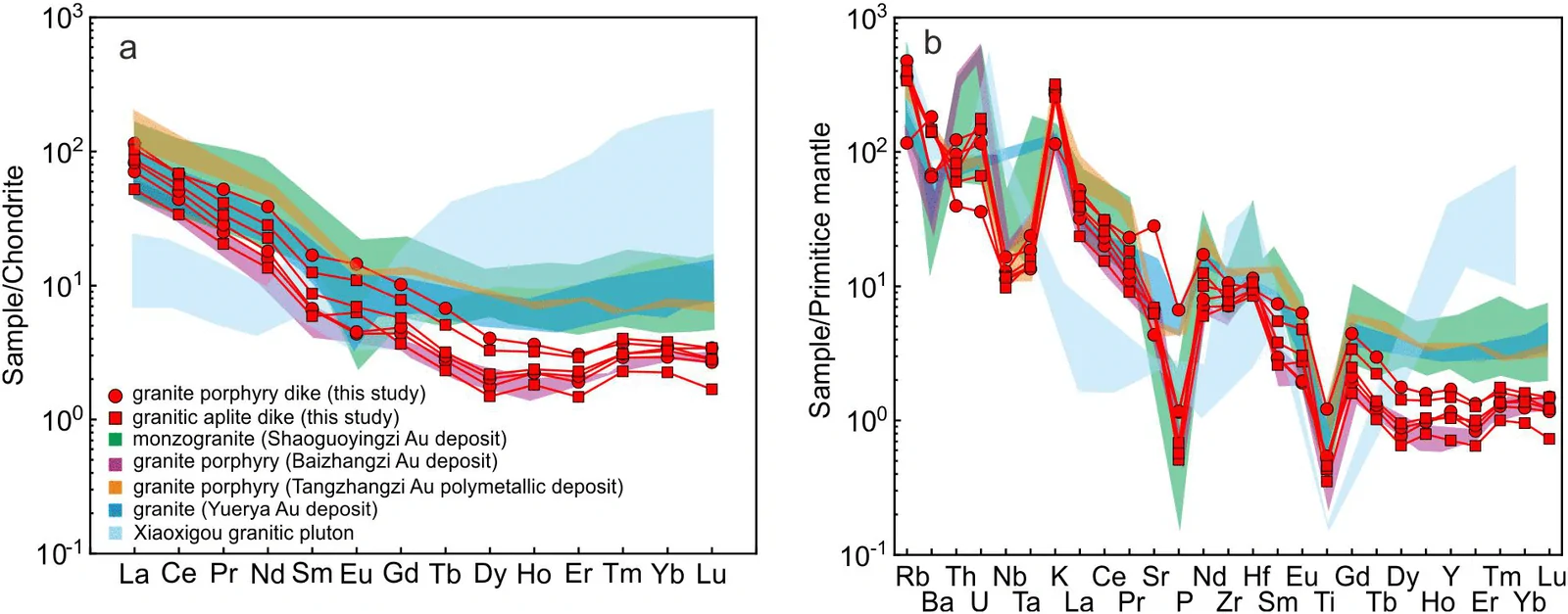
(a) Chondrite-normalized rare earth element patterns and (b) Primitive mantle-normalized trace element patterns for the granite porphyry dikes and granitic aplite dikes in the Xiaogushan gold deposit. The chondrite values are from Boynton, 1984 [54], and the primitive mantle values are from Sun and McDonough, 1989 [64]. The data for Shaoguoyingzi, Baizhangzi, Tangzhangzi, Yuerya and Xiaoxigou shown for comparison are from Chen et al. (2016) [29], Li et al. (2024) [18], He (2016) [30], Liu et al. (2015) [10] and Yang et al. (2019) [63], respectively.

The granitic aplite dikes show higher SiO_2_ contents (76.11 ~ 78.13 wt%) and total alkalis (Na_2_O+K_2_O = 9.87 ~ 10.25 wt%) compared to the granite porphyry dikes. All samples plot in the granite field (Fig 6a) and shoshonite series (Fig 6b), exhibiting peraluminous characteristics (A/CNK = 1.07 ~ 1.32; Al_2_O_3_=11.52 ~ 12.49 wt%) (Fig 6c). In the Na_2_O+K_2_O-CaO vs. SiO_2_ diagram (Fig 6d), they occupy transitional positions along the alkali-calcic to calc-alkaline boundary. The observation that the granitic aplite shows similar characteristics to the granite porphyry in both the chondrite-normalized REE patterns and the primitive mantle-normalized trace element patterns (Figs 7a and 7b).

## Discussion

### Intrusion timing of the dikes

Zircon U-Pb dating results show that the scatter off concordia, and the relatively high MSWD values (up to 5.1 and 9.7) indicate significant dispersion. All zircons used for age calculation exhibit magmatic Th/U ratios (>0.1) and oscillatory zoning in CL images, with no discernible inherited cores. The discordant analyses systematically plot below concordia line along a discordia trend, suggesting Pb loss rather than mixing with older components. Accordingly, the data dispersion is primarily attributed to variable Pb loss, a common phenomenon in LA‑ICP‑MS zircon U-Pb dating [65–68], potentially related to later hydrothermal alteration. The weighted mean ^206^Pb/^238^U ages obtained from 17 analyses of sample GPD‑2 (163.9 ± 2.0 Ma) and 7 analyses of sample GAD‑2 (162.2 ± 4.9 Ma) are consistent within analytical uncertainty. These ages are interpreted as the best estimates of the emplacement ages of the granite porphyry and granitic aplite dikes, respectively, constraining the magmatic activity to the Late Jurassic.

Mao et al. (2003) [69] reviewed the isotopic age data from both metal deposits and mineralized granitic rocks in North China and proposed that the three major mineralization periods were 110 ~ 130 Ma, 140 Ma and 160 ~ 200 Ma, which correspond to collisional orogenic processes, major changes in the tectonic system, and extensive lithospheric delamination, respectively. In addition, Qin et al. (2023) [70] proposed that the seven main metallogenic epochs of Au deposits in China are the Neoarchean-Paleoproterozoic (Wutai period), Mesoproterozoic-Neoproterozoic (Jinning-Xuefeng period), early Paleozoic (Caledonian period), late Paleozoic (Hercynian period), early Mesozoic (Indosinian period), late Mesozoic (Yanshanian period), and Cenozoic (Himalayan period). The Yanshanian period (late Mesozoic) represents the most significant gold mineralization event in China, exhibiting both the most extensive spatial distribution and highest abundance of gold deposits among all metallogenic epochs [70–72]. During this period, gold deposits were widely distributed across multiple metallogenic belts in China, with the most extensive occurrences concentrated along the northern margin and in the eastern regions of the North China Craton [70].

The eastern Hebei-western Liaoning region, located at the northern margin of the North China Craton, borders the southern segment of the prominent Yanshan orogenic belt. Geochronological data were compiled of magmatism and mineralization data in this region (Table 3; Fig 8). The area hosts numerous Mesozoic gold deposits accompanied by granitic plutons and intermediate-mafic dikes, including the representative Baizhangzi, Yuerya and Niuxinshan gold deposits [30,32,73–82]. As documented in Table 3 and Fig 8, Jurassic granites and volcanic rocks are widely distributed throughout the eastern Hebei-western Liaoning region, North China. The intrusive rocks were predominantly emplaced during 175 ~ 155 Ma (Middle to Late Jurassic). Numerous gold deposits spatially and temporally associated with these Jurassic intrusions occur across the region, displaying mineralization ages nearly identical to the magmatic events.

**Table 3:** Middle-Late Jurassic Geochronology of Magmatism and Mineralization in eastern Hebei-western Liaoning.

No.	Location/Deposit	Rock Type	Dated Material	Dating Method	Age (Ma)	Reference
Diagenetic age						
1	Tangzhangzi	Granite porphyry	Zircon	LA-ICP-MS	173.0 ± 5.5	[30]
2	Tangzhangzi	Diabase	Zircon	LA-ICP-MS	168.2 ± 1.7	[30]
3	Niuxinshan	Red granite	Zircon	SHRIMP	173 ± 2	[32]
4	Tangzhangzi	Granite porphyry	Zircon	SHRIMP	173 ± 2	[32]
5	Baizhangzi	Lamprophyre	Zircon	LA-ICP-MS	167 ± 8	[73]
6	Yuerya	Gray granite	Zircon	SHRIMP	175 ± 1	[74]
7	Yuerya	Meat red granite	Zircon	SHRIMP	174 ± 3	[74]
8	Jianchang	Quartz monzodiorite	Zircon	LA-ICP-MS	157 ± 1	[75]
9	Maanshan	Biotite hornblende monzonitic granite	Zircon	SHRIMP	155.9 ± 1.6	[76]
10	Maoshan	Monzogranite	Zircon	LA-ICP-MS	162.7 ± 1.5	[77]
11	Wangpingshi	Monzogranite	Zircon	LA-ICP-MS	162.3 ± 1.3	[77]
12	Gaojiadian	Quartz diorite	Zircon	LA-ICP-MS	170.5 ± 1.8	[77]
13	Xiaoyingzi	Granodiorite	Zircon	LA-ICP-MS	164.6 ± 2.2	[77]
14	Xiayingfang	Granite porphyry	Zircon	LA-ICP-MS	163.32 ± 0.9	[78]
15	Xiayingfang	Lamprophyre	Zircon	LA-ICP-MS	159.0 ± 1.5	[78]
16	Yuerya	Diorite	Zircon	LA-ICP-MS	163 ~ 155	[79]
17	Xiaogushan	Granite porphyry	Zircon	LA-ICP-MS	163.9 ± 2.0	This study
18	Xiaogushan	Granitic aplite	Zircon	LA-ICP-MS	162.2 ± 4.9	This study
Metallogenic age						
19	Xiaojiayingzi	Skarn	Molybdenite	Re-Os isotope	165.5 ± 4.6	[80]
20	Yuerya	Granite	Molybdenite	Re-Os isotope	169.84 ± 0.95	[81]
21	Tangzhangzi	Granite porphyry	Molybdenite	Re-Os isotope	170.0 ± 1.0	[82]
22	Xiayingfang	Lamprophyre	Molybdenite	Re-Os isotope	164.2 ± 2.3	[78]

**Fig 8:**
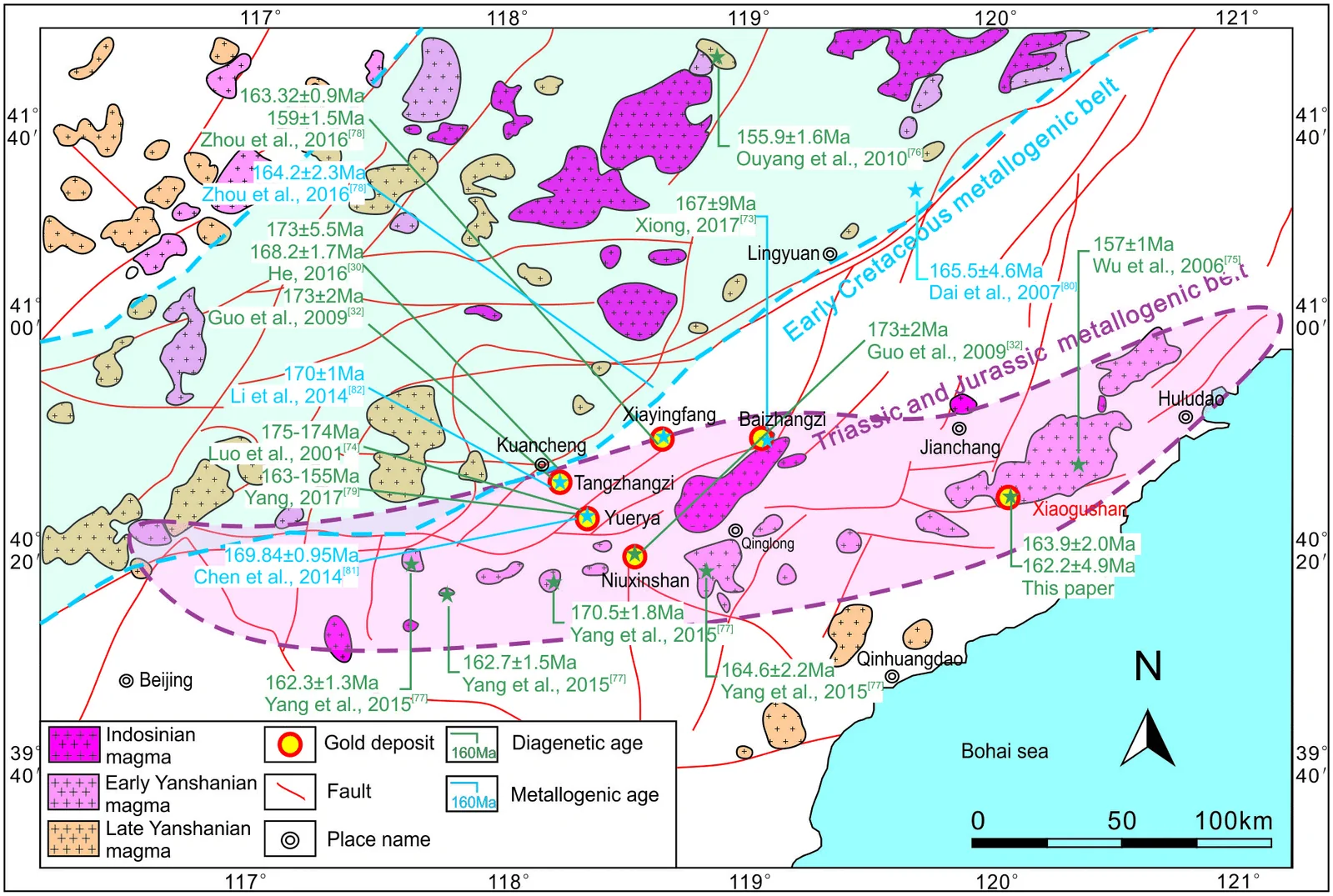
Spatial-temporal distributions of Late Jurassic igneous rocks and gold deposits in eastern Hebei-western Liaoning, China (modified after Xiong, 2017 [73]). Data for associated igneous rocks and mineralization ages of gold deposits are shown in Table 3.

Regional analysis indicates that the granitic dikes in the Xiaogushan gold deposit were emplaced during the Late Jurassic tectono-magmatic metallogenic event in the eastern Hebei-western Liaoning region. The formation of the deposit involved multiple episodes of hydrothermal, tectonic, and magmatic activity. The main mineralization stage is related to the Neoarchean cryptoexplosive breccia pipe. The mineralization enrichment and alteration features along the contacts of the Late Jurassic dikes suggest that this magmatic‑hydrothermal event may have superimposed and modified the earlier ore bodies. Consequently, the Late Jurassic dike activity represents the key geological process responsible for the later superimposed mineralization and ore remobilization, suggesting a multi-stage activity of ore-forming fluids in the area.

### Petrogenesis

#### Granite type.

Granites are commonly classified into M-type, A-type, I-type, or S-type based on their source characteristics and tectonic environments [83–88]. This classification scheme is used here to evaluate the genetic type of granitic dikes in the Xiaogushan gold deposit, which provides constraints on magma sources and tectonic setting.

The granitic dikes show clear crustal geochemical signatures, excluding an M-type origin derived from mantle magmas [89]. A-type granites are typically characterized by a high abundance of alkaline mafic minerals, which are absent in the studied samples based on mineralogical observations [85]. Geochemical discrimination further supports this interpretation. In the TFeO/MgO vs. Zr + Nb + Ce + Y diagram (Fig 9a) and TFeO/MgO vs. SiO_2_ diagram (Fig 9b), all samples plot in the field of I-type and S-type granites, thus excluding an A-type affinity. Their peraluminous nature (A/CNK = 1.07 ~ 1.32, avg. 1.17) and placement in the S-type field in (Na_2_O+K_2_O–CaO) vs. SiO_2_ and FAC diagrams (Figs 6c, 6d and 9c) support an S-type classification [18]. During early peraluminous magma evolution, Y was preferentially incorporated into Y-rich minerals [90]. In the Y vs. Rb discrimination diagram (Fig 9d), the samples exhibit a negative Y-Rb correlation, consistent with the evolutionary trend of S-type granites, unlike the positive correlation typical of I-type granites.

**Fig 9:**
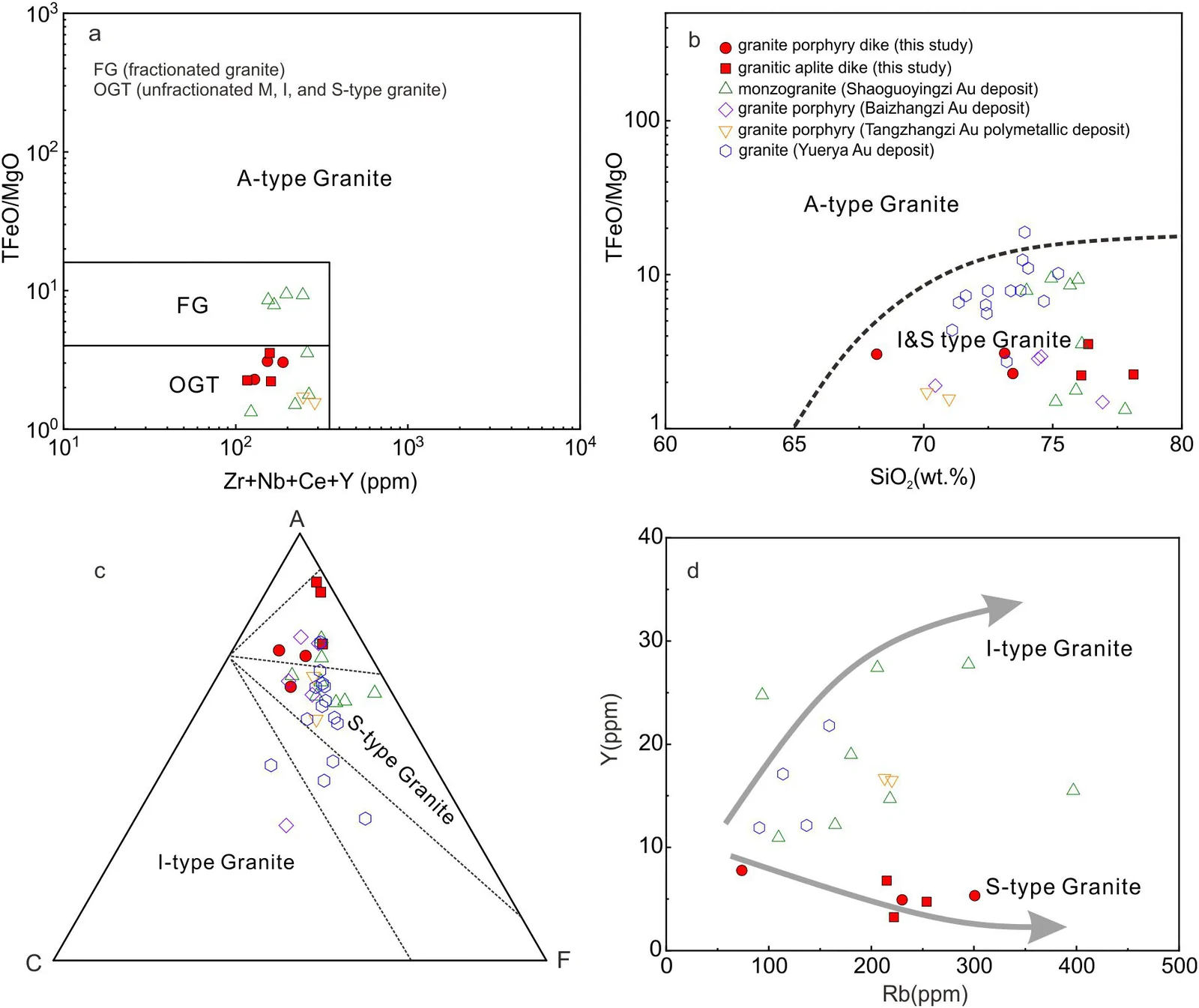
Discrimination diagrams of granite types for the granite porphyry dikes and granitic aplite dikes in the Xiaogushan gold deposit. (a) TFeO/MgO vs. Zr + Nb + Ce + Y (background field from Whalen et al., 1987 [85]), (b) TFeO/MgO vs. SiO_2_ (background field from Eby., 1990 [87]), (c) FAC (background field from White., 1977 [88]), (d) Y vs. Rb. The data for Shaoguoyingzi, Baizhangzi, Tangzhangzi and Yuerya shown for comparison are from Chen et al. (2016) [29], Li et al. (2024) [18], He (2016) [30], Liu et al. (2015) [10], respectively. F = MgO + FeO, A = Al_2_O_3_−Na_2_O−K_2_O, C = CaO.

These characteristics indicate that the granitic dikes in the Xiaogushan gold deposit are S‑type granites, which constrains regional magma genesis and crustal evolution, and accounts for the remobilization of Archean orebodies by Late Jurassic hydrothermal fluids. Furthermore, the close overlap of the Xiaogushan samples with regional granitic intrusions in Fig 9 points to a unified Late Jurassic tectono‑thermal event that triggered extensive crustal anatexis. The occurrence of I‑type affinities in a few intrusions implies a mixed source or minor mantle input, which may have supplied additional heat and volatiles, underscoring the complexity of crust‑mantle interaction. This further supports the interpretation that a multi‑stage, fluid‑rich magmatic system drove the late superimposed mineralization.

#### Magma source.

The granitic dikes in the Xiaogushan gold deposit display high SiO_2_, low Mg#, elevated K_2_O, enrichment in LILEs (e.g., Th, U, K), and depletion in HFSEs (e.g., Nb, Ta, P), suggesting a crustal source derived from partial melting of continental material [86,91–95]. Trace-element ratios such as Nb/Ta and Zr/Hf are geochemically stable and provide useful constraints of magma source characteristics [91,96]. Their measured ratios (Nb/Ta: 9.44 ~ 15.39, avg. 13.06; Zr/Hf: 27.08 ~ 40.27, avg. 31.16) and comparable to average continental crust values(Nb/Ta ≈ 11.4, Zr/Hf ≈ 33.33) [91], and differ from primitive mantle values (Nb/Ta ≈ 17.8, Zr/Hf ≈ 37) [64], further consistent with a crustal origin.

S-type granites originate from the anatexis of metasedimentary rocks, and their source characteristics can be evaluated using the Rb-Sr-Ba system [97]. In the Rb/Ba vs. Rb/Sr plot (Fig 10a), most samples plot near the clay-poor/clay-rich boundary, supporting a crustal origin. The granites can be genetically classified based on Sr and Yb concentrations, as these elements in intermediate-acid magmatic rocks reflect magma generation depth and source characteristics [94,98,101]. In the Sr vs. Yb diagram (Fig 10b), all samples plot within field II (low-Sr, low-Yb type), suggesting a medium- to low-pressure origin. The systematic variation from normal crust (Sr < 400 ppm, Yb > 2 ppm) to thickened crust (Sr < 100 ppm, Yb < 2 ppm) in the Xiaogushan area reveals an ongoing crustal thickening process. On the (La/Yb)_N_ vs. Yb_N_ (Fig 10c) and Sr/Y vs. Y (Fig 10d) diagrams, the samples plot in the lower part of the adakitic field, exhibiting moderate to slightly elevated (La/Yb)_N_ and Sr/Y ratios, but with consistently low Sr contents, which may be related to plagioclase fractionation during magma evolution. Although these geochemical features partially overlap with the adakitic field, the low Sr contents distinguish samples from typical slab-derived adakites, and instead make them more comparable to products of partial melting of thickened lower continental crust. These features are interpret as reflecting an adakitic like signature derived from partial melting of thickened ancient lower crust, which is consistent with the Late Jurassic continental thickening event in the northern margin of the North China Craton [102,103].

**Fig 10:**
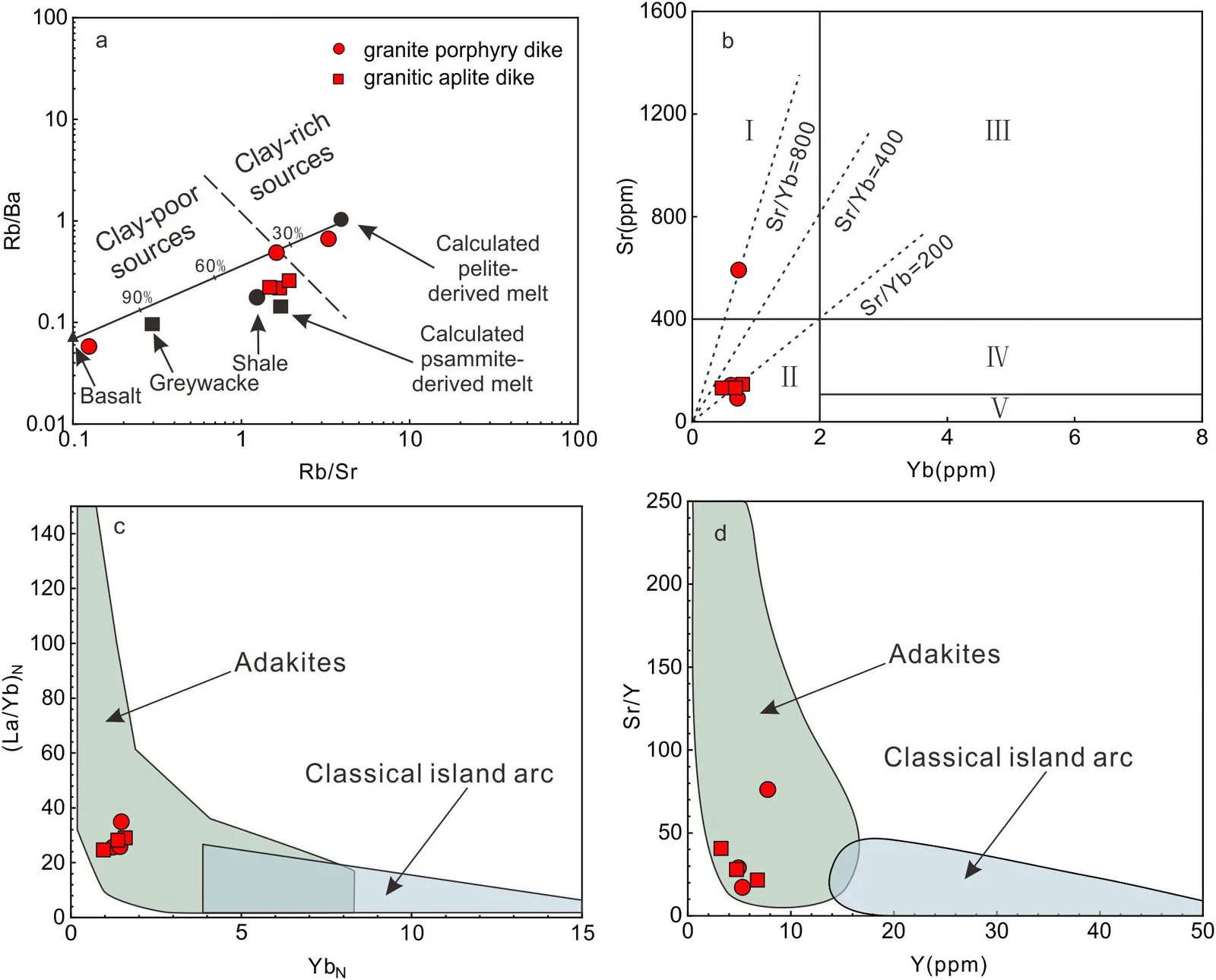
Plots of (a) Rb/Ba vs. Rb/Sr (after Sylvester, 1998 [97]), (b) Sr vs. Yb (after Zhang et al., 2006 [98]), (c) (La/Yb)_N_ vs. Yb_N_ (after Martin, 1986 [99]), and (d) Sr/Y vs. Y (after Drummond and Defant, 1990 [100]) for granite porphyry dikes and granitic aplite dikes in Xiaogushan gold deposit.

Zircon is physically and chemically robust, preserving its initial Hf isotopic compositions, and its low Lu content allows accurate Hf isotope determination at the time of crystallization [104–106], making it a powerful tracer of magma sources and petrogenesis. Positive εHf(t) values typically suggest derivation from depleted mantle or juvenile crust, whereas negative values indicate reworking of ancient crust [107,108]. In this study, zircons from the Xiaogushan dikes yield consistently negative εHf(t) values ranging from −17.66 to −13.47 (avg. −15.62) with T_DM2_ ages of 2333 ~ 2066 Ma (avg. 2201 Ma). In εHf(t) vs. age diagram (Fig 11a), all sample plot between the chondrite and 2.5 Ga crustal evolution lines, indicating a significant contribution from late Paleoproterozoic crust. In the ^176^Hf/^177^Hf vs. age diagram (Fig 11b), the data further confirm a lower-crustal source.

**Fig 11:**
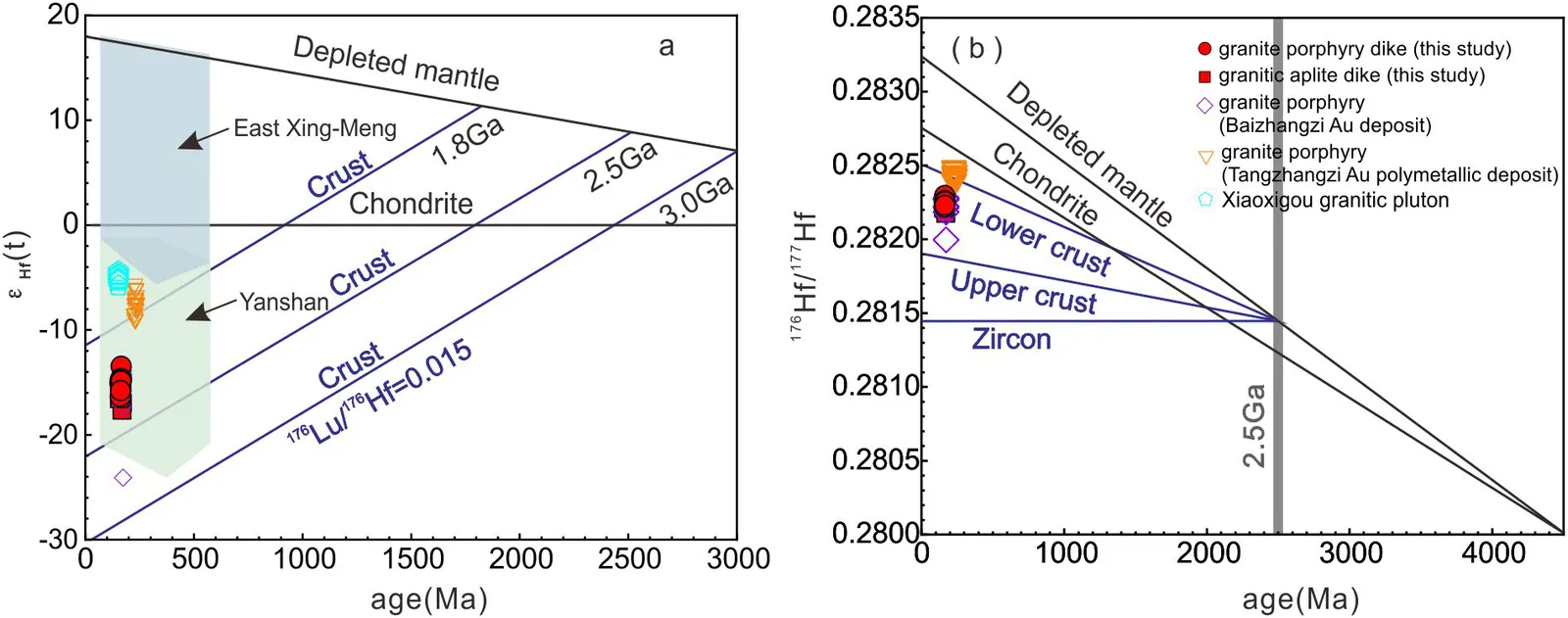
(a) εHf(t) vs. age (Ma) (after Yang et al., 2006 [109]) and (b) ^176^Hf/^177^Hf vs. age (Ma) (after Wu et al., 2007 [107]) diagrams for granite porphyry dikes and granitic aplite dikes in Xiaogushan gold deposit. The data for Baizhangzi, Tangzhangz and Xiaoxigou shown for comparison are from Li et al. (2024) [18], He (2016) [30] and Yang et al. (2019) [63], respectively.

The Hf isotopic signatures of the Xiaogushan granitic dikes show consistently negative εHf(t) values and Paleoproterozoic T_DM2_ ages, supporting an ancient lower‑crustal origin. Their broad overlap with regional intrusions, despite slightly more negative εHf(t), suggests melting of a common, homogeneous Paleoproterozoic crustal source during the Late Jurassic tectono‑magmatic event. Thus, the dikes formed by partial melting of Paleoproterozoic lower crust, reflecting widespread crustal reworking in the eastern Hebei-western Liaoning region at that time.

#### Magma evolution.

Granite is a major component of the continental crust, and its magmatic evolution has long been a key research focus in the geosciences [94]. The La/Sm vs. La diagram is widely used to distinguish between fractional crystallization and partial melting [110]. The samples define a trend consistent with fractional crystallization (Fig 12a), indicating that the evolution of the granitic magmas in this area was dominated by fractional crystallization. The Ba vs. Sr (Fig 12b) and Rb/Ba vs. Eu diagrams (Fig 12c) further confirm the fractionation of K-feldspar and plagioclase. The samples exhibit right‑inclined REE patterns with variable δEu values (0.79 ~ 1.35) mostly close to unity, suggesting limited plagioclase fractionation and variations among different dike types or evolutionary stages. Some samples with δEu > 1 (e.g., 1.35) may reflect plagioclase accumulation, whereas the weak negative anomalies are likely related to the difficulty of Eu^3+^ incorporation into plagioclase under relatively high oxygen fugacity. In addition, the Harker diagrams (Figs 12d ~ i) show negative correlations of Al_2_O_3_, MgO, TFe_2_O_3_, P_2_O_5_ and TiO_2_ with SiO_2_, and a positive correlation of K_2_O with SiO_2_, further supporting the dominant role of fractional crystallization in the magma evolution.

**Fig 12:**
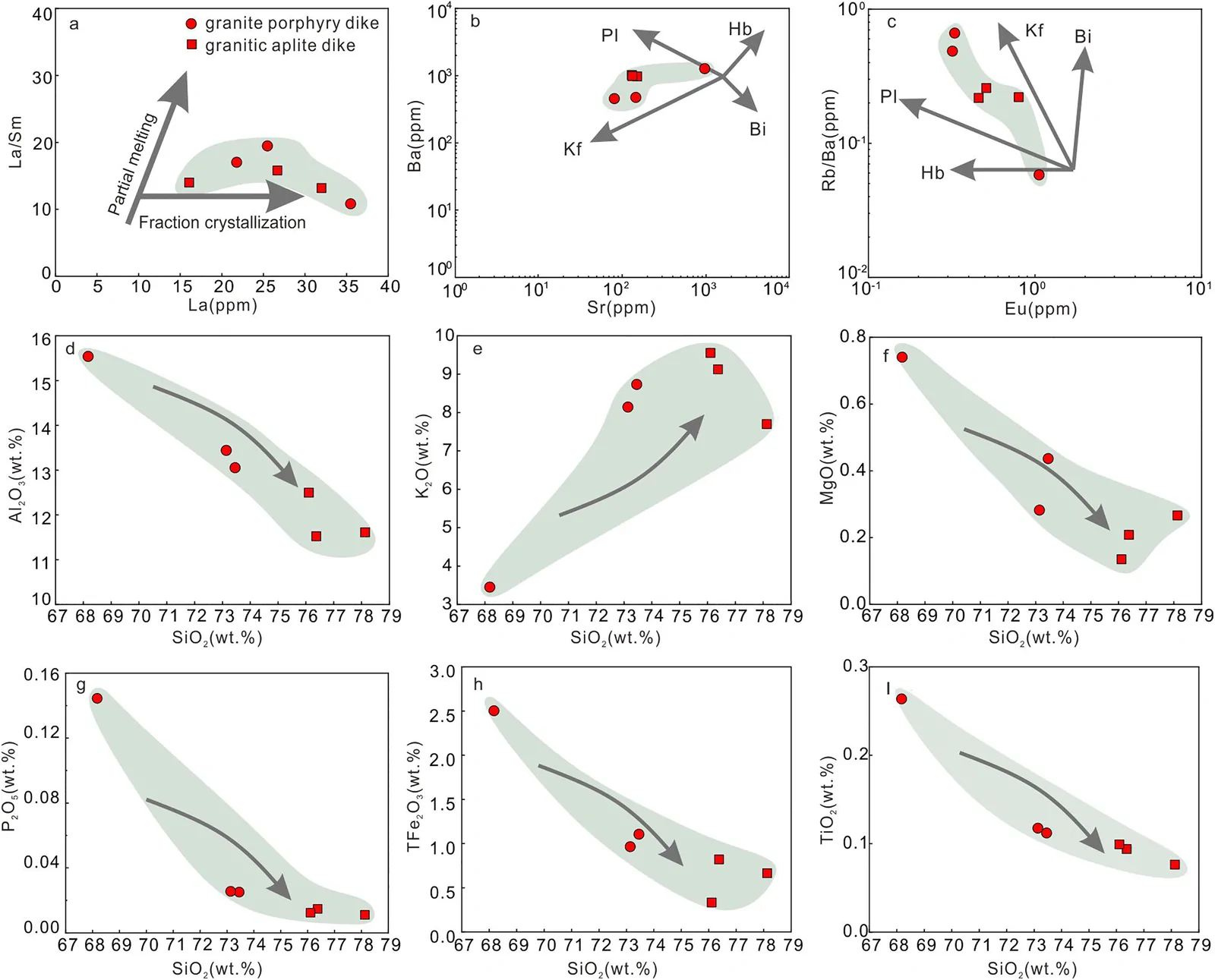
Plots of (a) La/Sm vs. La, (b) Ba vs. Sr (after Wang et al., 2013 [111]), (c) Rb/Ba vs. Eu, and Harker diagrams of (d) Al_2_O_3_ vs. SiO_2_, (e) K_2_O vs. SiO_2_, (f) MgO vs. SiO_2_, (g) P_2_O_5_ vs. SiO_2_, (h) Fe_2_O_3_ vs. SiO_2_, and (i) TiO_2_ vs. SiO_2_ for granite porphyry dikes and granitic aplite dikes in Xiaogushan gold deposit. The geochemical trends in (b-c) are consistent with fractional crystallization of K-feldspar and plagioclase during magma evolution. Pl, Kf, Bi and Hb denote the removal of plagioclase, K-feldspar, biotite and hornblende, respectively, during magma evolution.

### Tectonic setting

At present, there is controversy regarding the regional metallogenic tectonic background of the Middle to Late Jurassic period in the eastern Hebei-western Liaoning region. One view suggests that the Mesozoic in this area was affected by the subduction of the Paleo-Pacific Plate [19,112–114], while another proposes that this area was influenced by the Mongol-Okhotsk tectonic system [115–117]. The subduction of the Paleo-Pacific Plate beneath Eurasia during the Mesozoic Era progressed through three primary stages: the Early to Middle Jurassic, the late Early Cretaceous, and the Late Cretaceous, with the late Middle Jurassic to Early Cretaceous representing an intermittent period of subduction [115,116,118]. The Mongol-Okhotsk suture zone formed during the Middle Jurassic, characterized by a scissor-like closure of the Mongol-Okhotsk Ocean from west to east. The western segment closed during the Middle to Late Jurassic, whereas the eastern segment was finally closed in the Early Cretaceous [115,116,119–122].

During the Mesozoic era, the Mongol-Okhotsk tectonic regime significantly influenced the eastern Hebei-western Liaoning region, inducing multiple collisional events and consequent crustal thickening [123–126]. Li et al. (2015) [127] posited that a continental crust thickening event occurred in relation to the closure of the Mongol-Okhotsk suture zone, extending from the northwestern Lesser Khingan Range to northern Hebei-western Liaoning during the Late Jurassic, based on their study of late Jurassic muscovite granite in the Sunwu area. In the northern Hebei-western Liaoning area, a north-south trending thrust structure was identified within the Haifanggou Formation. The thrust nappe event occurred at 170 Ma, forming in response to crustal thickening following the closure of the Mongol-Okhotsk Ocean [128]. Ouyang et al. (2013) [129] proposed that the Late Jurassic mineralization in Northeast China and adjacent regions developed in a postorogenic extensional setting following the closure of the Mongol-Okhotsk Ocean.

Zircon U-Pb geochronology constrains the emplacement of granitic dikes in the Xiaogushan gold deposit to ca. 163.9 ~ 162.2 Ma. The studied samples exhibit strongly peraluminous S-type granite characteristics, which are typically associated with continental collision orogenic settings [130]. This interpretation is further corroborated by tectonic discrimination diagrams (Fig 13): in both the Rb vs. Y + Nb (Fig 13a) and 3Ta vs. Rb/10 vs. Hf (Fig 13b) plots, the majority of samples from the Xiaogushan deposit and adjacent areas consistently plot within the syn-COLG field. A few analyses extend into the VAG and WPG fields, which may reflect either a magmatic signature inherited from an earlier subduction‑related stage or the onset of post‑collisional extension, respectively. This coherent regional distribution supports a Late Jurassic continental collisional tectonic setting as a widespread regional feature, rather than a localized phenomenon. Geochemical signatures further indicate a source derived from thickened lower crust, suggesting these intrusions formed during syn‑ to late‑collisional crustal thickening following closure of the Mongol-Okhotsk Ocean.

**Fig 13:**
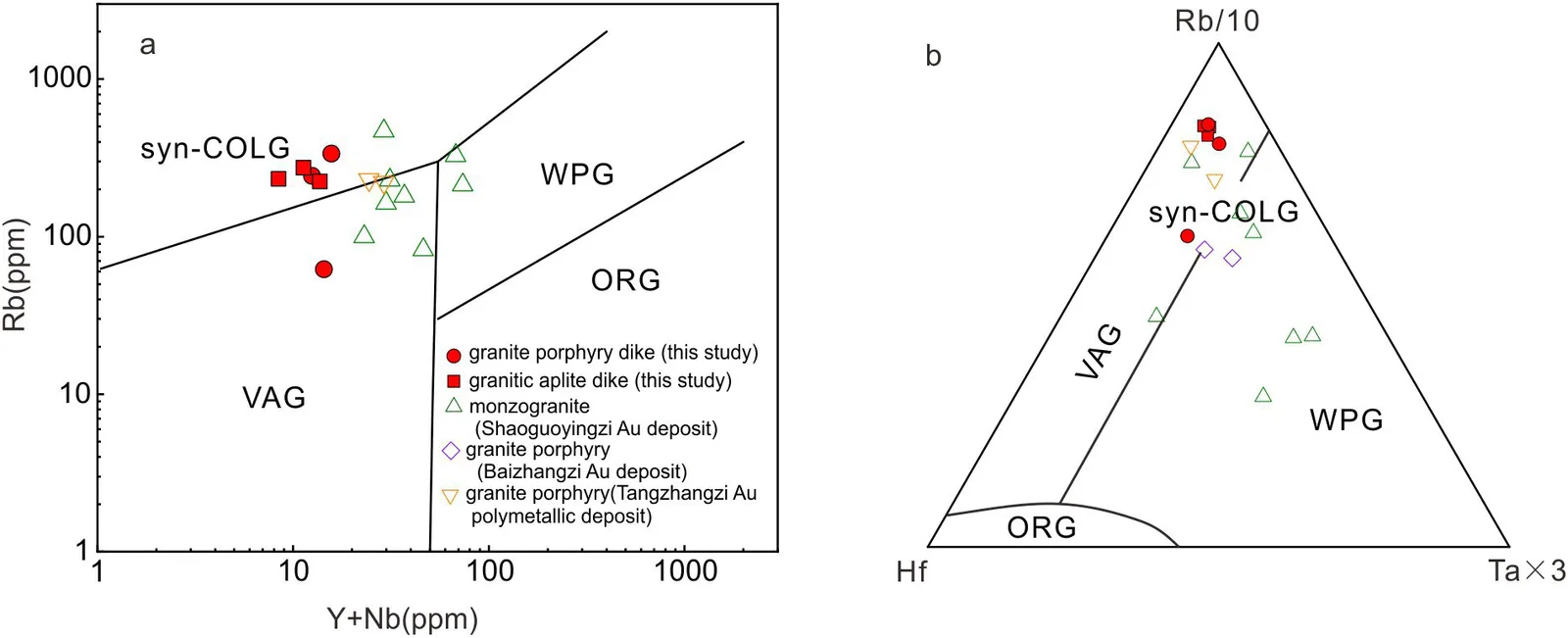
Tectonic discrimination diagrams of (a) Rb vs. Y + Nb (after Pearce et al., 1984 [131]) and (b) Ta × 3 vs. Rb/10 vs. Hf (after Harris et al., 1986 [132]) for granite porphyry dikes and granitic aplite dikes in Xiaogushan gold deposit. The abbreviations used in the figure are as follows: VAG, volcanic arc granite; ORG, ocean ridge granite; WPG, within-plate granite; and syn-COLG, syncollisional granite. The data for Shaoguoyingzi, Baizhangzi and Tangzhangzi shown for comparison are from Chen et al. (2016) [29], Li et al. (2024) [18], He (2016) [30], respectively.

In summary, the Late Jurassic granitic dikes in Xiaogushan gold deposit were formed in response to a continental collision setting associated with closure of the Mongol-Okhotsk suture zone. We therefore propose a tectonic model that constrains the Mesozoic evolution of the Mongol‑Okhotsk Ocean and its influence on the eastern Hebei-western Liaoning area (Fig 14). During the Triassic to Early Jurassic, the Mongol-Okhotsk oceanic plate underwent bidirectional subduction (northward and southward), generating intense and widespread magmatic activity along both margins of the tectonic belt [133–135] (Fig 14a). By the Middle Jurassic to Early Cretaceous, the Mongol-Okhotsk Ocean had completely closed, resulting in collision of the flanking terranes and ultimate formation of the Mongol-Okhotsk tectonic belt [119,136,137] (Fig 14b). During this period, magmatic activity diminished with localized development of S-type granites, represented by the Late Jurassic S-type granitic dikes in the Xiaogushan gold deposit, which were likely emplaced in a syn- to late-collisional tectonic setting. Furthermore, crustal thickening, deformation, and subsequent extension induced by the collision event facilitated mineralization in the region.

**Fig 14:**
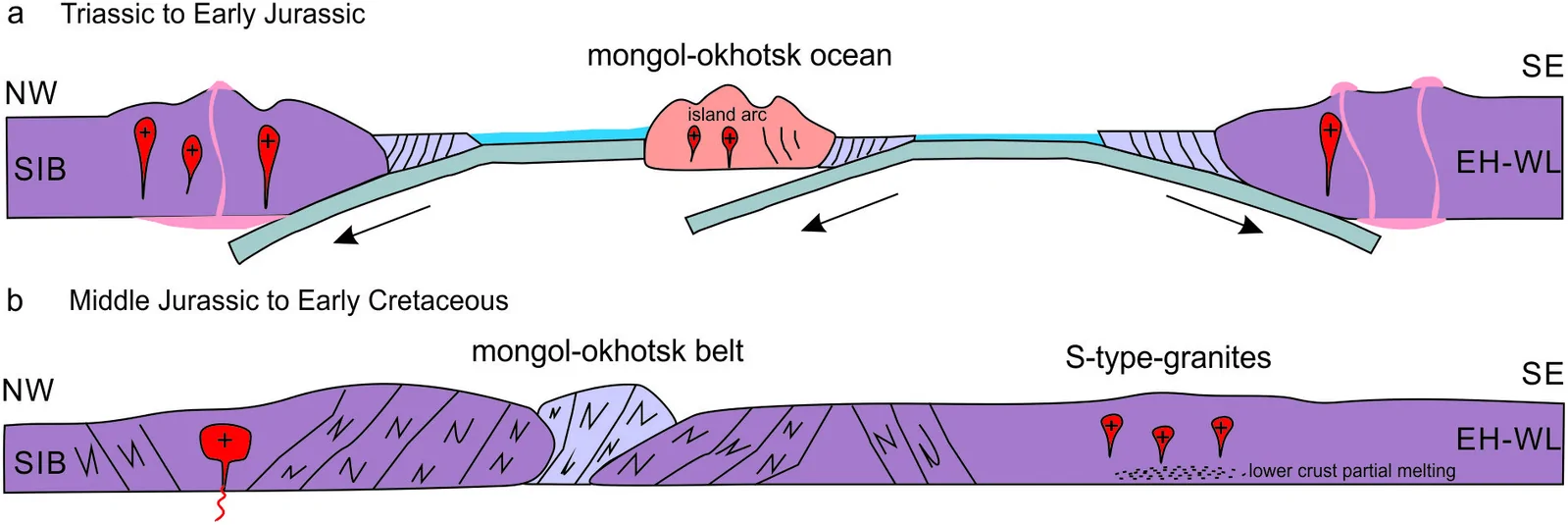
Simplified tectonic evolution model of the Mongol-Okhotsk Ocean and eastern Hebei-western Liaoning during the Mesozoic (modified after Huang et al., 2016 [133]; Liang et al., 2019 [134]). The abbreviations shown in the figure are as follows: SIB, Siberia Block; EH-WL, eastern Hebei-western Liaoning region.

### Implications for the Au mineralization potential

The metallogenic potential of granite can be evaluated through a framework that considers its oxidation state, compositional characteristics, extent of compositional evolution, and extent of fractionation [138–140].

The oxygen fugacity (*f*O_2_) of magmatic-hydrothermal systems is a key parameter controlling metal transport and mineralization potential. Studies have shown that the solubility of Au in hydrothermal fluids increases significantly with higher *f*O_2_ and temperature [141–145], indicating that high-oxygen fugacity environments favor the transport and enrichment of gold. Consequently, oxygen fugacity serves as a critical indicator for assessing metallogenic potential. Based on iron mineralogy, granitic rocks are commonly classified into oxidized magnetite series and reduced ilmenite series, with a well-established genetic link between magnetite-series granitoids and Au, Cu, and Mo deposits [35,146–148].

The studied samples are geochemically distinguished by high SiO_2_, low TiO_2_, and enriched HFSEs (Zr, Nb, Ta, Hf). Their elevated Fe_2_O_3_/FeO ratios (1.25 ~ 1.87) and position in the strongly oxidized (SO) field of the redox classification diagram (Fig 15a) confirm their affinity to the magnetite series [146,150]. Zircon oxygen fugacity (log*f*O_2_), calculated via Ti-in-zircon thermometry [143], reveals a systematic contrast: the granite porphyry crystallized under higher *f*O_2_ conditions (mean logfO_2_ = −8.28, ΔFMQ = +8.06, ΔNNO = +7.52) than the granitic aplite (mean logfO_2_ = −11.8, ΔFMQ = +3.58, ΔNNO = +3.03), suggesting a more favorable setting for gold mineralization (S2 Table). In the log*f*O_2_ vs. T_Ti_ diagram (Fig 15b), data from both dikes plot between the NNO and MH buffers, confirming a high-oxygen fugacity state for the entire magmatic system. This NNO-MH range promotes sulfate stabilization and low sulfur solubility in the melt, creating optimal conditions for the effective pre-ore retention of gold in the magma [138,141,151].

**Fig 15:**
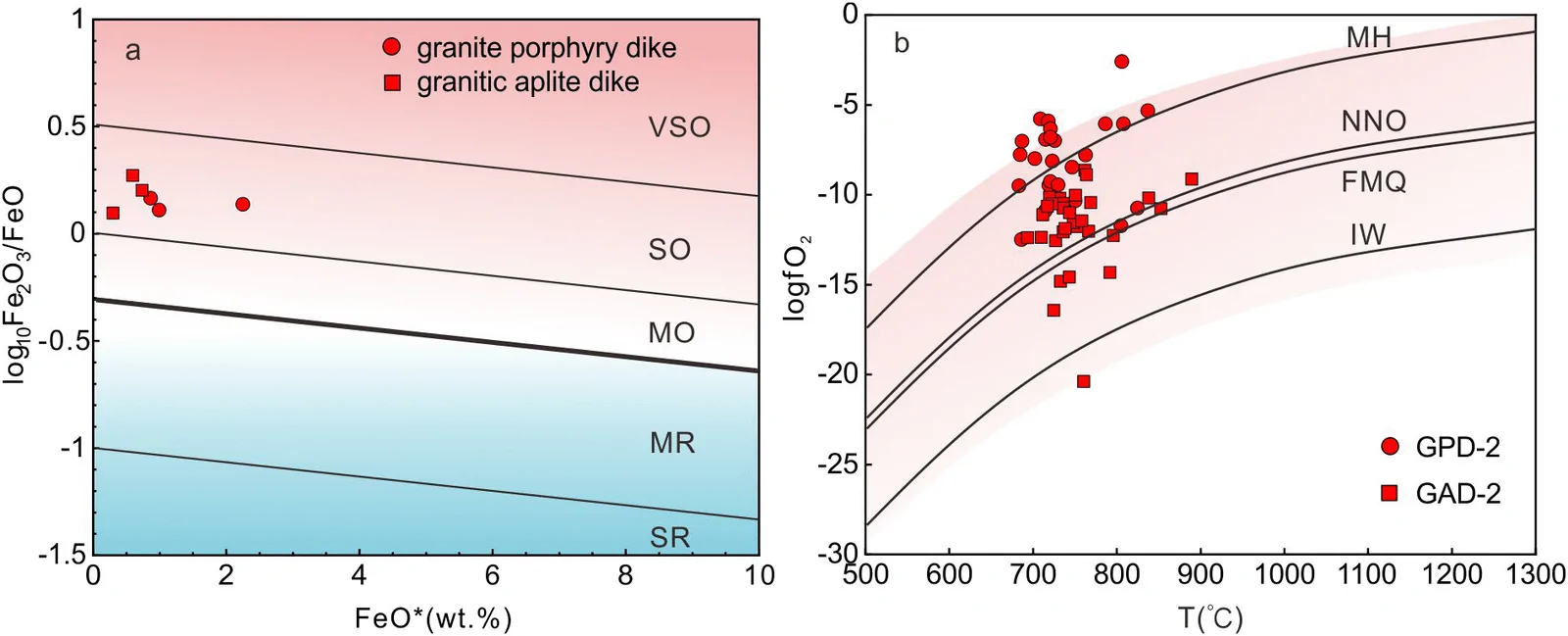
Plot of (a) log_10_Fe_2_O_3_/FeO vs. FeO* (after Blevin, 2004 [138]) and (b) logfO_2_ vs. T (°C) (after Qiu et al., 2013 [149]) for granite porphyry dikes and granitic aplite dikes in Xiaogushan gold deposit. Abbreviations shown in the figure are as follows: VSO, very strongly oxidized; SO, strongly oxidized; MO, moderately oxidized; MR, moderately reduced; SR, strongly reduced; MH, magnetite-hematite; NNO, nickel-nickel ore; FMQ, fayalite-magnetite-quartz; IW, iron-wustite. FeO* refers to all Fe in the sample reported as FeO.

Furthermore, key parameters such as aluminum saturation index, alkalinity, and SiO_2_ content critically govern the mineralogy and ore-forming element behavior of fractionating granitoids [138]. The alkali and K-rich dikes at Xiaogushan thus provide favorable geochemical conditions for gold enrichment and transport [36,152]. Magma evolution, as reflected by K/Rb ratios, further constrains metallogenic potential: granites are classified as unevolved (K/Rb > 400), moderately evolved (200 ~ 400), or strongly evolved (<200), with Cu-Au mineralized systems typically exhibiting ratios > 200 [138]. In this study, both the granite porphyry and granitic aplite dikes have K/Rb ratios exceeding 200 (averages of 309 and 317, respectively), indicating a moderately evolved magmatic system, which may be compatible with mineralized systems. This moderate evolution, together with active fractional crystallization as previously discussed, may have facilitated the effective remobilization and local redistribution of pre‑existing gold in the Xiaogushan system.

In summary, the granitic dikes in the Xiaogushan gold deposit exhibit geochemical characteristics of alkali enrichment, high oxygen fugacity, and moderate evolution, which favor hydrothermal fluid activity and local remobilization of pre-existing gold. Integrated geochronological and regional tectonic evolution analyses indicate that these dikes formed during the Late Jurassic tectono-magmatic-metallogenic period in the eastern Hebei-western Liaoning region. Their emplacement and associated hydrothermal systems have the potential to remobilize early ore-forming materials and promote superimposed mineralization at structurally favorable locations such as contact zones, while also possibly contributing some metallogenic components themselves [35]. Therefore, this magmatic-hydrothermal activity can be regarded as a potential key geological process driving late-stage superimposed mineralization and modification of the deposit, providing important insights into regional multi-stage fluid activities. Furthermore, such dikes, occurring in clusters within the region, can serve as effective prospecting indicators.

To better constrain the genetic relationship between diking and gold mineralization, future work should include high-precision geochronology of hydrothermal minerals (e.g., sericite, pyrite) integrated with fluid inclusion studies and isotopic tracing. Such approaches would help establish whether the spatial association reflects a true “temporally consistent and source-related” genetic link.

## Conclusion

This study culminated in the following conclusions.

(1) Zircon U-Pb dating constrains the emplacement of granite porphyry and granitic aplite dikes in the Xiaogushan gold deposit to 163.9 ± 2.0 Ma and 162.2 ± 4.9 Ma, respectively, confirming Late Jurassic magmatism in the eastern Hebei-western Liaoning region. This magmatic event may have provided a key driver for later-stage reactivation, modification, and superimposed mineralization of the deposit, thereby providing important evidence for the multi‑stage evolution of ore‑forming fluids in the region.
(2) The granite porphyry and granitic aplite dikes in the Xiaogushan gold deposit are strongly peraluminous S‑type granites belonging to the high‑K calc‑alkaline series. The primary magma was generated by partial melting of Paleoproterozoic lower crustal material. The magma underwent varying degrees of fractional crystallization of K-feldspar and plagioclase during its evolution.
(3) Late Jurassic magmatic activity in the eastern Hebei-western Liaoning region occurred within a continental collisional environment during the closure of the Mongolia-Okhotsk suture zone. This collisional event triggered crustal thickening, deformation, and eventual extension, which collectively facilitated mineralization in the region.
(4) The granitic dikes in the Xiaogushan gold deposit are geochemically characterized by alkali enrichment, high oxygen fugacity, and moderate evolution, which may be conducive to metal transport. Their associated hydrothermal systems have the potential to remobilize pre‑existing mineralization and promote further gold enrichment. Consequently, the clustered occurrence of such dikes in the region can serve as an effective prospecting indicator for deep‑seated hydrothermal activity and mineralizing potential.

## Supporting information

S1 Table LA-ICP-MS zircon U-Pb analysis data for granite porphyry dikes and granitic aplite dikes in Xiaogushan gold deposit. (DOCX)

S2 Table Trace elements compositions (ppm) of the zircons from granite porphyry dikes and granitic aplite dikes in Xiaogushan gold deposit. (DOCX)
