# Supplementary material for: Geochronology and geochemistry of granitic dikes in the Xiaogushan gold deposit NE China and implications for gold mineralization
Source: PLoS One. 2026 Oct 14;21(10):e0345886. doi: 10.1371/journal.pone.0345886 (PMC13667826; doi:10.1371/journal.pone.0345886)
Supplement: Supplementary file 1 [file pone.0345886.s001.docx]

Table 1. LA-ICP-MS zircon U-Pb analysis data for granite porphyry dikes and granitic aplite dikes in Xiaogushan gold deposit

| Sample  No. | Element content | | | Th/U | Isotopic ratios | | | | | | Age（Ma） | | | | | |
| --- | --- | --- | --- | --- | --- | --- | --- | --- | --- | --- | --- | --- | --- | --- | --- | --- |
|  | Pb  ppm | Th  ppm | U  ppm |  | ^207^Pb/^206^Pb | | ^207^Pb/^235^U | | ^206^Pb/^238^U | | ^207^Pb/^206^Pb | | ^207^Pb/^235^U | | ^206^Pb/^238^U | |
|  |  |  |  |  | Ratio | 1σ | Ratio | 1σ | Ratio | 1σ | Age | 1σ | Age | 1σ | Age | 1σ |
| GPD-2 (granite porphyry dikes) | | | | | | | | | | | | | | | | |
| 1 | 120 | 3529 | 2028 | 1.74 | 0.0530 | 0.0008 | 0.1764 | 0.0030 | 0.0241 | 0.0003 | 328 | 31 | 165 | 3 | 154 | 2 |
| 2 | 33.3 | 782 | 627 | 1.25 | 0.0514 | 0.0013 | 0.1787 | 0.0049 | 0.0251 | 0.0002 | 257 | 59 | 167 | 4 | 160 | 2 |
| 3* | 51.4 | 1013 | 904 | 1.12 | 0.0628 | 0.0021 | 0.2374 | 0.0072 | 0.0275 | 0.0003 | 702 | 71 | 216 | 6 | 175 | 2 |
| 4 | 38.2 | 730 | 828 | 0.88 | 0.0508 | 0.0011 | 0.1827 | 0.0039 | 0.0261 | 0.0003 | 232 | 54 | 170 | 3 | 166 | 2 |
| 5 | 28.2 | 526 | 689 | 0.76 | 0.0511 | 0.0014 | 0.1832 | 0.0052 | 0.0259 | 0.0003 | 256 | 60 | 171 | 4 | 165 | 2 |
| 6* | 38.3 | 744 | 647 | 1.15 | 0.0674 | 0.0018 | 0.2526 | 0.0070 | 0.0271 | 0.0003 | 850 | 56 | 229 | 6 | 172 | 2 |
| 7 | 77.4 | 1882 | 1236 | 1.52 | 0.0537 | 0.0012 | 0.1946 | 0.0042 | 0.0262 | 0.0003 | 367 | 48 | 181 | 4 | 167 | 2 |
| 8* | 20.9 | 402 | 356 | 1.13 | 0.0630 | 0.0019 | 0.2390 | 0.0065 | 0.0277 | 0.0004 | 707 | 64 | 218 | 5 | 176 | 2 |
| 9 | 58.3 | 1267 | 1174 | 1.08 | 0.0502 | 0.0010 | 0.1779 | 0.0032 | 0.0257 | 0.0003 | 206 | 44 | 166 | 3 | 163 | 2 |
| 10 | 38.7 | 734 | 815 | 0.90 | 0.0519 | 0.0013 | 0.1936 | 0.0047 | 0.0271 | 0.0003 | 280 | 56 | 180 | 4 | 172 | 2 |
| 11* | 65.5 | 1407 | 1076 | 1.31 | 0.0608 | 0.0013 | 0.2243 | 0.0049 | 0.0267 | 0.0003 | 632 | 44 | 206 | 4 | 170 | 2 |
| 12 | 71.4 | 1596 | 1340 | 1.19 | 0.0532 | 0.0011 | 0.1897 | 0.0040 | 0.0258 | 0.0002 | 345 | 48 | 176 | 3 | 164 | 1 |
| 13* | 111 | 2615 | 1805 | 1.45 | 0.0655 | 0.0016 | 0.2364 | 0.0075 | 0.0258 | 0.0004 | 791 | 50 | 215 | 6 | 165 | 2 |
| 14* | 9.10 | 143 | 175 | 0.82 | 0.0765 | 0.0033 | 0.3221 | 0.0159 | 0.0300 | 0.0004 | 1107 | 85 | 284 | 12 | 191 | 3 |
| 15 | 71.2 | 1475 | 1619 | 0.91 | 0.0508 | 0.0011 | 0.1780 | 0.0040 | 0.0254 | 0.0003 | 232 | 45 | 166 | 3 | 162 | 2 |
| 16 | 74.1 | 1572 | 1348 | 1.17 | 0.0524 | 0.0012 | 0.1939 | 0.0050 | 0.0267 | 0.0003 | 302 | 50 | 180 | 4 | 170 | 2 |
| 17 | 51.3 | 1121 | 983 | 1.14 | 0.0578 | 0.0013 | 0.2049 | 0.0046 | 0.0257 | 0.0003 | 524 | 48 | 189 | 4 | 163 | 2 |
| 18 | 50.6 | 1302 | 834 | 1.56 | 0.0518 | 0.0012 | 0.1810 | 0.0040 | 0.0253 | 0.0003 | 280 | 47 | 169 | 3 | 161 | 2 |
| 19 | 41.4 | 825 | 901 | 0.92 | 0.0506 | 0.0012 | 0.1817 | 0.0043 | 0.0260 | 0.0003 | 233 | 54 | 170 | 4 | 165 | 2 |
| 20* | 32.2 | 711 | 462 | 1.54 | 0.0587 | 0.0016 | 0.2166 | 0.0064 | 0.0267 | 0.0004 | 554 | 59 | 199 | 5 | 170 | 2 |
| 21 | 104 | 2888 | 1859 | 1.55 | 0.0488 | 0.0009 | 0.1669 | 0.0038 | 0.0247 | 0.0004 | 200 | 47 | 157 | 3 | 157 | 2 |
| 22 | 76.1 | 1776 | 1437 | 1.24 | 0.0503 | 0.0009 | 0.1787 | 0.0035 | 0.0256 | 0.0002 | 209 | 43 | 167 | 3 | 163 | 1 |

Table 1. (continued)

| Sample  No. | Element content | | | Th/U | Isotopic ratios | | | | | | Age（Ma） | | | | | |
| --- | --- | --- | --- | --- | --- | --- | --- | --- | --- | --- | --- | --- | --- | --- | --- | --- |
|  | Pb  ppm | Th  ppm | U  ppm |  | ^207^Pb/^206^Pb | | ^207^Pb/^235^U | | ^206^Pb/^238^U | | ^207^Pb/^206^Pb | | ^207^Pb/^235^U | | ^206^Pb/^238^U | |
|  |  |  |  |  | Ratio | 1σ | Ratio | 1σ | Ratio | 1σ | Age | 1σ | Age | 1σ | Age | 1σ |
| 23 | 35.1 | 870 | 562 | 1.55 | 0.0511 | 0.0015 | 0.1832 | 0.0051 | 0.0261 | 0.0003 | 243 | 69 | 171 | 4 | 166 | 2 |
| 24 | 71.7 | 1668 | 1281 | 1.30 | 0.0559 | 0.0011 | 0.2016 | 0.0042 | 0.0261 | 0.0003 | 450 | 47 | 186 | 4 | 166 | 2 |
| 25* | 34.8 | 448 | 353 | 1.27 | 0.1734 | 0.0093 | 0.6717 | 0.0438 | 0.0279 | 0.0006 | 2590 | 90 | 522 | 27 | 177 | 4 |
| 26* | 52.9 | 1271 | 752 | 1.69 | 0.0740 | 0.0019 | 0.2741 | 0.0060 | 0.0270 | 0.0003 | 1043 | 51 | 246 | 5 | 172 | 2 |
| GAD-2 (granitic aplite dikes) | | | | | | | | | | | | | | | | |
| 1 | 15.5 | 330 | 316 | 1.04 | 0.0500 | 0.0016 | 0.1851 | 0.0058 | 0.0269 | 0.0003 | 195 | 108 | 172 | 5 | 171 | 2 |
| 2 | 17.6 | 385 | 319 | 1.21 | 0.0518 | 0.0017 | 0.1905 | 0.0060 | 0.0267 | 0.0003 | 280 | 69 | 177 | 5 | 170 | 2 |
| 3* | 29.5 | 583 | 447 | 1.30 | 0.0735 | 0.0019 | 0.2658 | 0.0074 | 0.0261 | 0.0003 | 1028 | 53 | 239 | 6 | 166 | 2 |
| 4* | 17.1 | 377 | 330 | 1.14 | 0.0612 | 0.0020 | 0.2133 | 0.0069 | 0.0252 | 0.0003 | 656 | 70 | 196 | 6 | 161 | 2 |
| 5 | 23.2 | 561 | 433 | 1.29 | 0.0567 | 0.0016 | 0.1958 | 0.0056 | 0.0250 | 0.0002 | 480 | 61 | 182 | 5 | 159 | 1 |
| 6* | 55.1 | 626 | 519 | 1.21 | 0.2156 | 0.0082 | 0.7536 | 0.0248 | 0.0255 | 0.0003 | 2948 | 62 | 570 | 14 | 162 | 2 |
| 7 | 33.1 | 886 | 564 | 1.57 | 0.0539 | 0.0016 | 0.1857 | 0.0053 | 0.0250 | 0.0003 | 369 | 65 | 173 | 5 | 159 | 2 |
| 8* | 46.5 | 366 | 413 | 0.89 | 0.2175 | 0.0085 | 0.8554 | 0.0399 | 0.0280 | 0.0004 | 2962 | 63 | 628 | 22 | 178 | 2 |
| 9* | 23.9 | 308 | 308 | 1.00 | 0.1227 | 0.0052 | 0.4663 | 0.0206 | 0.0275 | 0.0006 | 1998 | 75 | 389 | 14 | 175 | 4 |
| 10* | 21.6 | 404 | 356 | 1.13 | 0.0791 | 0.0031 | 0.2813 | 0.0107 | 0.0258 | 0.0003 | 1176 | 78 | 252 | 8 | 164 | 2 |
| 11* | 66.8 | 834 | 582 | 1.43 | 0.2333 | 0.0075 | 0.8387 | 0.0312 | 0.0258 | 0.0003 | 3076 | 46 | 618 | 17 | 164 | 2 |
| 12* | 34.1 | 486 | 428 | 1.14 | 0.1324 | 0.0048 | 0.4705 | 0.0168 | 0.0260 | 0.0006 | 2129 | 63 | 392 | 12 | 165 | 4 |
| 13* | 59.7 | 852 | 616 | 1.38 | 0.2064 | 0.0111 | 0.6552 | 0.0306 | 0.0237 | 0.0004 | 2877 | 87 | 512 | 19 | 151 | 2 |
| 14* | 57.3 | 508 | 552 | 0.92 | 0.2270 | 0.0152 | 0.7483 | 0.0494 | 0.0242 | 0.0003 | 3031 | 107 | 567 | 29 | 154 | 2 |
| 15* | 30.9 | 493 | 432 | 1.14 | 0.1060 | 0.0030 | 0.4215 | 0.0105 | 0.0290 | 0.0004 | 1732 | 52 | 357 | 7 | 184 | 2 |
| 16 | 24.1 | 574 | 458 | 1.25 | 0.0508 | 0.0016 | 0.1792 | 0.0055 | 0.0256 | 0.0002 | 232 | 68 | 167 | 5 | 163 | 2 |
| 17* | 23.6 | 458 | 415 | 1.10 | 0.0803 | 0.0029 | 0.2839 | 0.0115 | 0.0254 | 0.0003 | 1206 | 72 | 254 | 9 | 162 | 2 |
| 18 | 24.0 | 538 | 486 | 1.11 | 0.0541 | 0.0017 | 0.1937 | 0.0058 | 0.0262 | 0.0003 | 376 | 77 | 180 | 5 | 166 | 2 |

Table 1. (continued)

| Sample  No. | Element content | | | Th/U | Isotopic ratios | | | | | | Age（Ma） | | | | | |
| --- | --- | --- | --- | --- | --- | --- | --- | --- | --- | --- | --- | --- | --- | --- | --- | --- |
|  | Pb  ppm | Th  ppm | U  ppm |  | ^207^Pb/^206^Pb | | ^207^Pb/^235^U | | ^206^Pb/^238^U | | ^207^Pb/^206^Pb | | ^207^Pb/^235^U | | ^206^Pb/^238^U | |
|  |  |  |  |  | Ratio | 1σ | Ratio | 1σ | Ratio | 1σ | Age | 1σ | Age | 1σ | Age | 1σ |
| 19* | 20.9 | 455 | 392 | 1.16 | 0.0730 | 0.0031 | 0.2400 | 0.0097 | 0.0239 | 0.0004 | 1013 | 86 | 218 | 8 | 152 | 2 |
| 20* | 26.3 | 291 | 311 | 0.94 | 0.3538 | 0.0487 | 1.8487 | 0.2583 | 0.0369 | 0.0022 | 3724 | 210 | 1063 | 92 | 234 | 14 |
| 21* | 52.4 | 370 | 397 | 0.93 | 0.3022 | 0.0123 | 1.0475 | 0.0428 | 0.0252 | 0.0004 | 3483 | 63 | 728 | 21 | 160 | 3 |
| 22* | 21.2 | 329 | 322 | 1.02 | 0.0903 | 0.0046 | 0.3388 | 0.0186 | 0.0269 | 0.0004 | 1431 | 96 | 296 | 14 | 171 | 2 |
| 23* | 18.7 | 423 | 355 | 1.19 | 0.0683 | 0.0026 | 0.2365 | 0.0092 | 0.0250 | 0.0003 | 880 | 84 | 216 | 8 | 159 | 2 |
| 24* | 35.4 | 565 | 430 | 1.31 | 0.1447 | 0.0044 | 0.4917 | 0.0145 | 0.0247 | 0.0004 | 2284 | 52 | 406 | 10 | 157 | 3 |
| 25* | 28.4 | 517 | 436 | 1.19 | 0.0822 | 0.0025 | 0.2945 | 0.0088 | 0.0260 | 0.0002 | 1251 | 59 | 262 | 7 | 165 | 2 |
| 26 | 24.9 | 652 | 462 | 1.41 | 0.0527 | 0.0015 | 0.1783 | 0.0049 | 0.0246 | 0.0002 | 322 | 63 | 167 | 4 | 157 | 2 |
| 27* | 32.4 | 706 | 487 | 1.45 | 0.0914 | 0.0075 | 0.3033 | 0.0235 | 0.0241 | 0.0003 | 1455 | 153 | 269 | 18 | 154 | 2 |
| 28* | 53.9 | 717 | 508 | 1.41 | 0.1897 | 0.0071 | 0.6884 | 0.0262 | 0.0263 | 0.0003 | 2740 | 95 | 532 | 16 | 167 | 2 |
| 29* | 33.9 | 394 | 380 | 1.04 | 0.1658 | 0.0041 | 0.5759 | 0.0157 | 0.0251 | 0.0003 | 2517 | 42 | 462 | 10 | 160 | 2 |
| 30* | 23.0 | 431 | 424 | 1.02 | 0.0679 | 0.0030 | 0.2319 | 0.0088 | 0.0255 | 0.0014 | 878 | 92 | 212 | 7 | 163 | 9 |

Note: The sample data of label * is the data removed when calculating the weighted value.
