# Supplementary material for: Geochronology and geochemistry of granitic dikes in the Xiaogushan gold deposit NE China and implications for gold mineralization
Source: PLoS One. 2026 Oct 14;21(10):e0345886. doi: 10.1371/journal.pone.0345886 (PMC13667826; doi:10.1371/journal.pone.0345886)
Supplement: Supplementary file 2 [file pone.0345886.s002.docx]

Table 2. Trace elements compositions (ppm) of the zircons from granite porphyry dikes and granitic aplite dikes in Xiaogushan gold deposit

| Sample  No. | La | Ce | Pr | Nd | Sm | Eu | Gd | Tb | Dy | Ho | Er | Tm | Yb | Lu | δEu | δCe | logfO2 | △FMQ | △NNO |
| --- | --- | --- | --- | --- | --- | --- | --- | --- | --- | --- | --- | --- | --- | --- | --- | --- | --- | --- | --- |
| GPD-2 (granite porphyry dikes) | | | | | | | | | | | | | | | | | | | |
| 1 | 0.23 | 189 | 0.41 | 4.06 | 7.14 | 1.53 | 36.3 | 11.9 | 153 | 59.9 | 306 | 74.6 | 804 | 145 | 0.29 | 148.32 | -8.13 | 7.97 | 7.39 |
| 2 | 0.38 | 91.1 | 0.17 | 1.89 | 2.86 | 1.34 | 15.9 | 5.18 | 68.0 | 29.5 | 160 | 40.5 | 510 | 93.4 | 0.61 | 86.24 | -8.00 | 8.64 | 8.05 |
| 3 | 0.15 | 90.5 | 0.13 | 1.82 | 3.21 | 0.74 | 17.9 | 6.69 | 93.8 | 38.8 | 218 | 53.7 | 648 | 121 | 0.30 | 151.97 | -2.60 | 11.58 | 11.05 |
| 4 | 9.06 | 118 | 2.16 | 9.98 | 4.12 | 1.29 | 19.5 | 6.78 | 99.7 | 43.8 | 243 | 65.3 | 829 | 161 | 0.44 | 6.42 | -10.89 | 5.42 | 4.84 |
| 5 | 0.12 | 60.0 | 0.12 | 1.05 | 2.11 | 0.55 | 11.1 | 3.97 | 62.5 | 28.0 | 174 | 47.5 | 611 | 125 | 0.35 | 123.10 | -5.79 | 10.68 | 10.09 |
| 6 | 20.7 | 164 | 6.24 | 26.7 | 11.6 | 2.75 | 38.7 | 11.6 | 149 | 54.1 | 263 | 61.7 | 685 | 122 | 0.40 | 3.47 | -10.75 | 3.04 | 2.52 |
| 7 | 0.42 | 138 | 0.43 | 4.63 | 8.05 | 2.37 | 33.4 | 10.6 | 133 | 53.6 | 289 | 69.5 | 853 | 156 | 0.44 | 77.47 | -8.48 | 7.04 | 6.48 |
| 8 | 0.31 | 72.5 | 0.11 | 1.46 | 2.54 | 0.98 | 14.0 | 4.54 | 61.5 | 24.0 | 128 | 31.6 | 373 | 69.4 | 0.51 | 93.80 | -9.51 | 7.63 | 7.03 |
| 9 | 0.047 | 129 | 0.12 | 1.68 | 3.76 | 0.77 | 24.0 | 8.54 | 124 | 52.8 | 289 | 72.2 | 868 | 156 | 0.25 | 411.25 | -7.03 | 10.01 | 9.42 |
| 10 | 0.21 | 91.9 | 0.15 | 1.67 | 3.06 | 0.91 | 17.3 | 5.92 | 90.5 | 41.5 | 236 | 60.3 | 758 | 147 | 0.38 | 124.29 | -5.90 | 10.32 | 9.74 |
| 11 | 0.38 | 127 | 0.37 | 3.68 | 5.83 | 1.15 | 29.2 | 9.93 | 126 | 48.9 | 253 | 58.8 | 679 | 117 | 0.27 | 80.89 | -6.06 | 8.54 | 8.00 |
| 12 | 0.26 | 143 | 0.23 | 2.63 | 4.18 | 1.39 | 23.6 | 8.57 | 115 | 48.4 | 271 | 69.2 | 827 | 158 | 0.43 | 140.35 | -6.34 | 9.82 | 9.24 |
| 13 | 0.97 | 176 | 1.32 | 9.31 | 7.92 | 1.80 | 28.8 | 9.05 | 116 | 47.8 | 260 | 65.2 | 801 | 143 | 0.36 | 37.45 | -5.34 | 8.19 | 7.68 |
| 14 | 0.13 | 52.8 | 0.061 | 1.33 | 2.73 | 1.31 | 13.6 | 4.36 | 53.9 | 20.5 | 102 | 23.2 | 262 | 48.6 | 0.66 | 144.45 | -9.48 | 6.72 | 6.14 |
| 15 | 39.3 | 333 | 23.3 | 100 | 21.3 | 2.78 | 35.9 | 9.75 | 130 | 53.6 | 301 | 77.1 | 983 | 188 | 0.31 | 2.65 | -15.37 | 16.09 | 16.05 |
| 16 | 0.45 | 152 | 0.34 | 3.27 | 4.71 | 1.24 | 22.9 | 7.69 | 119 | 50.4 | 283 | 73.7 | 922 | 176 | 0.37 | 92.70 | -6.93 | 9.38 | 8.80 |
| 17 | 0.54 | 112 | 0.54 | 4.95 | 5.14 | 1.08 | 23.3 | 7.40 | 104 | 42.3 | 231 | 57.7 | 683 | 124 | 0.30 | 49.88 | -7.80 | 7.34 | 6.79 |
| 18 | 0.048 | 135 | 0.15 | 2.55 | 4.35 | 1.92 | 25.0 | 8.15 | 108 | 42.8 | 223 | 54.9 | 649 | 120 | 0.56 | 387.55 | -6.99 | 9.04 | 8.47 |
| 19 | 5.19 | 111 | 1.56 | 7.98 | 4.88 | 1.22 | 25.1 | 8.68 | 121 | 51.7 | 283 | 73.9 | 913 | 175 | 0.34 | 9.40 | -12.50 | 4.56 | 3.96 |
| 20 | 0.20 | 84.1 | 0.35 | 3.32 | 3.97 | 1.45 | 18.7 | 5.33 | 71.8 | 28.6 | 145 | 34.6 | 402 | 77.0 | 0.51 | 76.51 | -6.05 | 8.08 | 7.55 |
| 21 | 0.0099 | 172 | 0.094 | 2.10 | 5.50 | 1.11 | 32.6 | 10.4 | 140 | 58.6 | 310 | 75.9 | 890 | 162 | 0.25 | 1355.4 | -7.77 | 9.33 | 8.73 |
| 22 | 0.30 | 160 | 0.41 | 3.69 | 4.68 | 1.44 | 24.8 | 8.34 | 119 | 53.9 | 297 | 76.4 | 945 | 174 | 0.41 | 111.15 | -6.81 | 9.34 | 8.76 |
| 23 | 0.15 | 99.7 | 0.19 | 2.65 | 5.67 | 2.49 | 31.8 | 11.4 | 150 | 57.4 | 277 | 64.2 | 691 | 123 | 0.57 | 142.22 | -9.27 | 6.89 | 6.31 |

Table 2. (continued)

| Sample  No. | La | Ce | Pr | Nd | Sm | Eu | Gd | Tb | Dy | Ho | Er | Tm | Yb | Lu | δEu | δCe | logfO2 | △FMQ | △NNO |
| --- | --- | --- | --- | --- | --- | --- | --- | --- | --- | --- | --- | --- | --- | --- | --- | --- | --- | --- | --- |
| 24 | 0.45 | 139 | 0.67 | 5.31 | 5.37 | 1.42 | 29.0 | 8.86 | 117 | 43.7 | 229 | 55.6 | 630 | 114 | 0.35 | 60.94 | -9.45 | 6.48 | 5.91 |
| 25 | 0.66 | 89.2 | 0.82 | 5.92 | 5.25 | 2.00 | 23.3 | 6.83 | 92.7 | 36.7 | 189 | 45.2 | 560 | 101 | 0.55 | 29.18 | -10.35 | 5.09 | 4.53 |
| 26 | 3.89 | 167 | 3.34 | 21.4 | 14.9 | 4.55 | 45.6 | 13.6 | 167 | 57.9 | 273 | 61.4 | 659 | 116 | 0.53 | 11.12 | -11.73 | 2.46 | 1.94 |
| GAD-2 (granitic aplite dikes) | | | | | | | | | | | | | | | | | | | |
| 1 | 7.51 | 102 | 1.99 | 10.1 | 5.90 | 2.17 | 27.6 | 8.62 | 113 | 42.3 | 209 | 45.9 | 515 | 95.2 | 0.52 | 6.37 | -14.82 | 1.04 | 0.47 |
| 2 | 14.0 | 115 | 3.79 | 16.0 | 6.52 | 1.98 | 23.3 | 7.41 | 93.1 | 35.5 | 177 | 39.2 | 438 | 82.5 | 0.49 | 3.81 | -16.43 | -0.38 | -0.95 |
| 3 | 0.19 | 105 | 0.15 | 2.35 | 4.90 | 2.06 | 27.4 | 8.54 | 113 | 41.4 | 204 | 45.8 | 509 | 94.7 | 0.54 | 151.64 | -10.20 | 5.68 | 5.11 |
| 4 | 2.06 | 99.2 | 0.61 | 4.76 | 4.85 | 2.09 | 24.6 | 8.24 | 107 | 41.2 | 204 | 46.4 | 511 | 96.1 | 0.59 | 21.30 | -10.44 | 4.55 | 4.00 |
| 5 | 0.035 | 119 | 0.13 | 2.34 | 4.67 | 2.08 | 28.9 | 9.71 | 124 | 46.3 | 234 | 51.7 | 565 | 105 | 0.55 | 427.01 | -10.15 | 6.04 | 5.46 |
| 7 | 4.71 | 114 | 2.44 | 13.3 | 10.5 | 4.41 | 33.4 | 10.1 | 126 | 44.8 | 227 | 50.9 | 568 | 105 | 0.72 | 8.10 | -10.79 | 2.43 | 1.92 |
| 8 | 2.68 | 133 | 0.83 | 5.18 | 6.04 | 2.37 | 31.9 | 9.85 | 123 | 45.0 | 209 | 45.8 | 493 | 92.7 | 0.52 | 21.46 | -12.57 | 3.43 | 2.86 |
| 9 | 0.96 | 111 | 0.79 | 5.79 | 6.40 | 2.71 | 31.0 | 9.83 | 137 | 50.0 | 249 | 54.8 | 613 | 115 | 0.59 | 30.46 | -11.79 | 3.58 | 3.03 |
| 10 | 0.24 | 88.3 | 0.26 | 2.63 | 4.36 | 2.04 | 24.6 | 7.88 | 106 | 41.4 | 206 | 45.5 | 528 | 98.2 | 0.60 | 84.57 | -10.50 | 5.27 | 4.70 |
| 11 | 0.22 | 99.4 | 0.24 | 2.93 | 5.54 | 2.20 | 28.5 | 9.22 | 117 | 44.1 | 220 | 48.3 | 543 | 99.8 | 0.54 | 103.12 | -12.38 | 4.06 | 3.48 |
| 12 | 2.40 | 135 | 1.87 | 18.2 | 12.9 | 5.33 | 46.0 | 13.3 | 159 | 58.6 | 279 | 58.6 | 626 | 116 | 0.67 | 15.39 | -14.32 | 0.16 | -0.37 |
| 13 | 1.10 | 120 | 0.53 | 4.80 | 6.71 | 2.73 | 33.2 | 10.4 | 128 | 48.7 | 237 | 52.1 | 575 | 105 | 0.56 | 37.55 | -11.57 | 3.92 | 3.36 |
| 14 | 9.99 | 128 | 2.43 | 14.0 | 10.2 | 3.81 | 35.4 | 10.9 | 131 | 48.5 | 233 | 50.8 | 565 | 105 | 0.61 | 6.25 | -9.13 | 3.38 | 2.90 |
| 15 | 2.36 | 122 | 1.65 | 10.8 | 8.99 | 4.06 | 37.6 | 11.4 | 145 | 53.4 | 256 | 57.8 | 634 | 117 | 0.67 | 14.83 | -10.18 | 3.32 | 2.81 |
| 16 | 0.12 | 114 | 0.26 | 2.58 | 4.97 | 2.18 | 28.0 | 9.21 | 124 | 43.7 | 227 | 48.9 | 551 | 103 | 0.57 | 156.02 | -11.12 | 5.27 | 4.68 |
| 17 | 0.62 | 123 | 0.28 | 3.14 | 4.93 | 2.14 | 29.9 | 9.35 | 125 | 47.4 | 229 | 51.4 | 562 | 104 | 0.54 | 71.93 | -12.40 | 4.47 | 3.87 |
| 18 | 11.3 | 139 | 2.90 | 14.1 | 7.19 | 2.55 | 29.2 | 9.17 | 115 | 42.1 | 203 | 45.8 | 508 | 95.0 | 0.54 | 5.86 | -14.59 | 1.01 | 0.45 |
| 19 | 0.58 | 111 | 0.71 | 4.76 | 5.70 | 2.27 | 27.4 | 8.87 | 118 | 43.0 | 214 | 46.9 | 525 | 97.5 | 0.56 | 41.71 | -12.06 | 3.72 | 3.15 |
| 20 | 0.51 | 110 | 0.32 | 2.95 | 5.55 | 1.88 | 30.5 | 9.22 | 118 | 46.8 | 218 | 48.5 | 540 | 97.8 | 0.44 | 65.36 | -10.75 | 5.03 | 4.46 |
| 21 | 0.15 | 90.3 | 0.24 | 3.04 | 4.47 | 1.87 | 22.1 | 7.60 | 102 | 40.1 | 209 | 47.0 | 514 | 98.6 | 0.58 | 112.19 | -10.03 | 5.40 | 4.84 |

Table 2. (continued)

| Sample  No. | La | Ce | Pr | Nd | Sm | Eu | Gd | Tb | Dy | Ho | Er | Tm | Yb | Lu | δEu | δCe | logfO2 | △FMQ | △NNO |
| --- | --- | --- | --- | --- | --- | --- | --- | --- | --- | --- | --- | --- | --- | --- | --- | --- | --- | --- | --- |
| 22 | 2.12 | 83.8 | 1.27 | 8.49 | 6.94 | 2.75 | 26.9 | 7.74 | 110 | 40.4 | 204 | 43.8 | 503 | 96.3 | 0.62 | 12.28 | -12.28 | 2.12 | 1.59 |
| 23 | 0.26 | 88.1 | 0.21 | 2.01 | 4.67 | 1.90 | 24.2 | 8.24 | 108 | 42.0 | 209 | 45.4 | 526 | 95.9 | 0.55 | 89.67 | -8.64 | 6.52 | 5.97 |
| 24 | 0.086 | 94.7 | 0.19 | 1.93 | 4.89 | 2.03 | 26.3 | 8.30 | 109 | 40.4 | 200 | 45.2 | 507 | 91.7 | 0.55 | 178.37 | -10.51 | 5.60 | 5.03 |
| 25 | 0.46 | 106 | 0.53 | 3.76 | 6.14 | 2.38 | 28.4 | 8.95 | 108 | 40.2 | 190 | 43.0 | 474 | 87.6 | 0.55 | 51.94 | -11.88 | 3.84 | 3.27 |
| 26 | 0.55 | 127 | 0.38 | 4.33 | 6.13 | 2.54 | 34.7 | 10.7 | 141 | 52.3 | 253 | 55.4 | 599 | 113 | 0.53 | 66.75 | -11.01 | 4.58 | 4.02 |
| 27 | 0.11 | 108 | 0.13 | 2.16 | 4.36 | 1.93 | 26.4 | 8.41 | 106 | 38.6 | 188 | 42.3 | 455 | 84.4 | 0.55 | 217.64 | -10.65 | 5.61 | 5.03 |
| 28 | 5.11 | 129 | 1.37 | 8.47 | 6.14 | 2.59 | 32.0 | 9.84 | 124 | 44.0 | 213 | 47.1 | 532 | 96.1 | 0.57 | 11.74 | -12.04 | 3.02 | 2.47 |
| 29 | 1592 | 2687 | 216 | 741 | 118 | 33.7 | 103 | 17.9 | 174 | 57.0 | 275 | 60.5 | 653 | 117 | 0.93 | 1.10 | -20.39 | -5.20 | -5.75 |
| 30 | 0.74 | 94.5 | 0.49 | 4.29 | 5.91 | 2.50 | 31.2 | 9.56 | 121 | 45.0 | 217 | 47.6 | 530 | 100 | 0.56 | 37.75 | -11.46 | 3.78 | 3.23 |
